# Citrus Flavonoids as Promising Phytochemicals Targeting Diabetes and Related Complications: A Systematic Review of In Vitro and In Vivo Studies

**DOI:** 10.3390/nu12102907

**Published:** 2020-09-23

**Authors:** Gopalsamy Rajiv Gandhi, Alan Bruno Silva Vasconcelos, Ding-Tao Wu, Hua-Bin Li, Poovathumkal James Antony, Hang Li, Fang Geng, Ricardo Queiroz Gurgel, Narendra Narain, Ren-You Gan

**Affiliations:** 1 Research Center for Plants and Human Health, Institute of Urban Agriculture, Chinese Academy of Agricultural Sciences (CAAS), Chengdu 600103, China; egarajiv@gmail.com (G.R.G.); tiantsai@sina.com (H.L.); 2 Chengdu National Agricultural Science and Technology Center, Chengdu 600103, China; 3 Postgraduate Program of Health Sciences (PPGCS), Federal University of Sergipe (UFS), Prof. João Cardoso Nascimento Campus, Aracaju, Sergipe 49060-108, Brazil; ricardoqgurgel@gmail.com; 4 Postgraduate Program of Physiological Sciences (PROCFIS), Federal University of Sergipe (UFS), Campus São Cristóvão, São Cristóvão, Sergipe 49100-000, Brazil; abs.vasconcelos@gmail.com; 5 Institute of Food Processing and Safety, College of Food Science, Sichuan Agricultural University, Ya’an 625014, China; DT_Wu@sicau.edu.cn; 6 Guangdong Provincial Key Laboratory of Food, Nutrition and Health, Department of Nutrition, School of Public Health, Sun Yat-sen University, Guangzhou 510080, China; lihuabin@mail.sysu.edu.cn; 7 Department of Microbiology, St. Xavier’s College, Kathmandu 44600, Nepal; jamesantonysj@gmail.com; 8 Key Laboratory of Coarse Cereal Processing (Ministry of Agriculture and Rural Affairs), School of Food and Biological Engineering, Chengdu University, Chengdu 610106, China; gengfang@cdu.edu.cn; 9 Laboratory of Flavor and Chromatographic Analysis, Federal University of Sergipe, Campus São Cristóvão, São Cristóvão, Sergipe 49.100-000, Brazil; narendra.narain@gmail.com

**Keywords:** citrus, diabetes, flavonoids, inflammation, polyphenols

## Abstract

The consumption of plant-based food is important for health promotion, especially concerning the prevention and management of chronic diseases. Flavonoids are the main bioactive compounds in citrus fruits, with multiple beneficial effects, especially antidiabetic effects. We systematically review the potential antidiabetic action and molecular mechanisms of citrus flavonoids based on in vitro and in vivo studies. A search of the PubMed, EMBASE, Scopus, and Web of Science Core Collection databases for articles published since 2010 was carried out using the keywords citrus, flavonoid, and diabetes. All articles identified were analyzed, and data were extracted using a standardized form. The search identified 38 articles, which reported that 19 citrus flavonoids, including 8-prenylnaringenin, cosmosiin, didymin, diosmin, hesperetin, hesperidin, isosiennsetin, naringenin, naringin, neohesperidin, nobiletin, poncirin, quercetin, rhoifolin, rutin, sineesytin, sudachitin, tangeretin, and xanthohumol, have antidiabetic potential. These flavonoids regulated biomarkers of glycemic control, lipid profiles, renal function, hepatic enzymes, and antioxidant enzymes, and modulated signaling pathways related to glucose uptake and insulin sensitivity that are involved in the pathogenesis of diabetes and its related complications. Citrus flavonoids, therefore, are promising antidiabetic candidates, while their antidiabetic effects remain to be verified in forthcoming human studies.

## 1. Introduction

The genus Citrus covers a large diversity of trees and shrubs, containing 16 species (according to Swingle’s classification) or 156 species (according to Tanaka’s classification), and is native to subtropical and tropical regions of Asia (from India to North China) and Oceania (Queensland and Australia) [1]. The high phenotypic and genetic variability of the Citrus genus is explained by the sexual compatibility between the Citrus species, allowing natural hybridization, and the long history of human intervention by interspecific hybridization to obtain more useful varieties of the plants [2]. The resulting hybrids are often considered as novel species, in spite of their ability to cross with each other [3]. Additionally, spontaneous natural mutations also increase the diversity of citrus varieties [4]. 

Citrus fruits are produced and consumed all over the world and represent an annual production of 100 million tons, with 60 million tons being consumed locally, 10 million tons exported, and 30 million tons used in industrial production [5]. The market is dominated by the production of oranges, lemons, limes, pomelos, grapefruit, mandarins, and their hybrids. However, there has recently been increasing consumption of uncommon citrus hybrids, such as yuzu, kaffir lime, blood oranges, and kumquats. The first identification of flavonoids in the Citrus genus dates back to the late nineteenth century when hesperidin was discovered by pioneering biochemistry work [6]. Since then, 44 flavonoids naturally presenting in citrus have been described [7]. Flavonoids are present in diverse citrus fruits, such as bergamots, grapefruits, lemons, limes, mandarins, oranges, and pomelos [8]. Citrus flavonoids include flavones, flavanones, flavonols, isoflavones, anthocyanidins, and flavanols [7]. Some of them are characteristic compounds of the genus, especially polymethoxyflavones (PMFs), while others may selectively present in certain varieties. 

Diabetes mellitus is a chronic disease causing 4.2 million deaths and an additional economic burden of 760 million US dollars in health expenditure around the globe in 2019 [9]. According to the latest diabetic data provided by the International Diabetic Federation (IDF), about 463 million adults aged between 20 and 79 years have diabetes, most of them living in low- and middle-income countries, and this is estimated to rise to 700 million by 2045 [9]. The disease is characterized by multiple serious health complications such as nephropathy, neuropathy, and retinopathy that can damage internal organs, particularly the pancreas, heart, liver, adipose tissue, and the kidneys, requiring comprehensive health care and management. It has also become the leading cause of various chronic metabolic diseases [10]. It occurs as a consequence of the irregular catabolism and anabolism of carbohydrates, lipids, and proteins, due to insulin resistance or hypoinsulinism [11]. Abnormal glycemic regulation may increase micro- and macro-vascular diseases, and impair vascular homeostasis. Furthermore, it is suggested that diabetic subjects have an increased risk of physical and cognitive disability, cancer, tuberculosis, and depression [12,13,14,15]). Although it is a multifactorial disease, some studies suggest that oxidative stress due to extreme hyperglycemia plays a pivotal role in the initiation of various pathological conditions, such as inflammation and atherosclerosis [15,16].

Many flavonoids derived from citrus fruits have been reported to reduce oxidative stress, improve glucose tolerance and insulin sensitivity, modulate lipid metabolism and adipocyte differentiation, suppress inflammation and apoptosis, and improve endothelial dysfunction [8,17,18,19,20,21,22], which indicates their potential antidiabetic effect. In order to highlight the antidiabetic potential of citrus flavonoids, we carried out a systematic review to provide related evidence in vitro and in vivo based on the PRISMA (Preferred Reporting Items for Systematic Reviews and Meta-Analyses) guidelines [23]. The article search was conducted in March 2020, and the survey covered articles published since 2010. The search was performed in four databases, including PubMed, EMBASE, Scopus and the Web of Science Core Collection, using the keywords “flavonoid”, “citrus”, and “diabetes”. 

**Figure 1:**
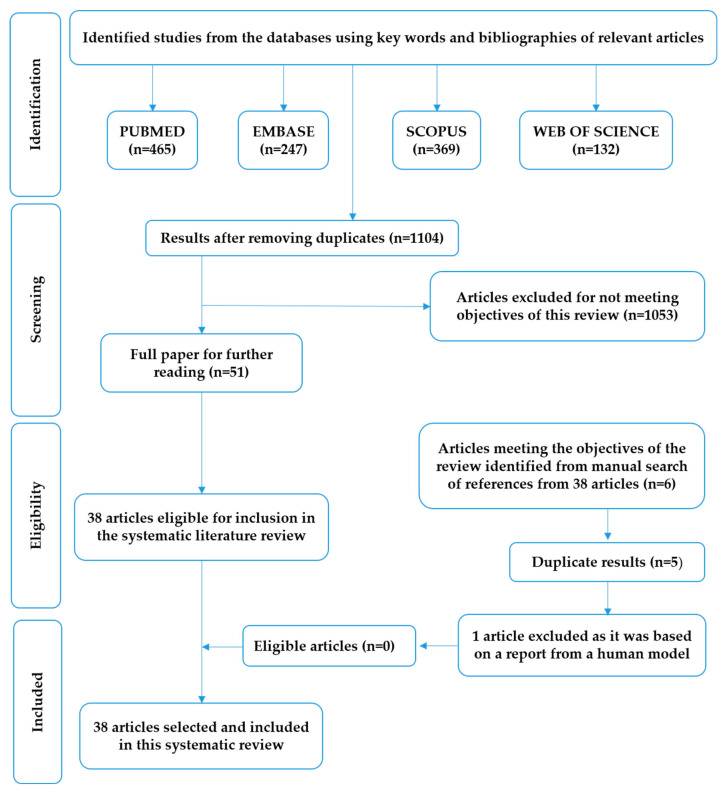
The flowchart of study selection for this systematic review.

The initial search found 1213 articles in the databases (PubMed: 465, EMBASE: 369, Scopus: 247, and Web of Science: 132). The inclusion criteria were in vitro and in vivo studies of citrus flavonoids, articles in English, and published between 2010 and March 2020. The exclusion criteria were articles not in English, human studies, reviews, letters, commentaries, editorials, and case reports. Following the application of the inclusion and exclusion criteria, and after discarding any duplication, we collected 38 articles that contained studies discussing the pharmacological activity of 19 flavonoids of the genus Citrus in relation to diabetes. The flowchart of study selection for this systematic review is provided in Figure 1. This review paper mainly summarizes the antidiabetic potential of the main citrus flavonoids based on in vitro and in vivo studies, and discusses related mechanisms of action of citrus flavonoids.

## 2. Main Citrus Flavonoids with Antidiabetic Effects

**Figure 2:**
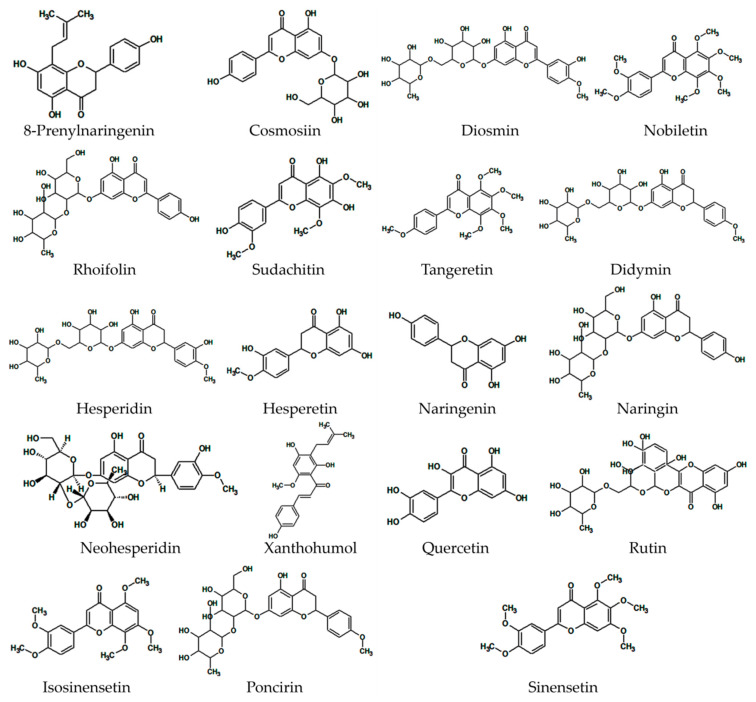
The 19 main citrus flavonoids with antidiabetic effects summarized from 38 articles.

The 19 citrus flavonoids discussed are presented in Figure 2. The following section summarizes the main antidiabetic flavonoids, discussing their antidiabetic effects and related mechanisms based on in vitro (Table 1) and in vivo (Table 2) studies. 

### 2.1. 8-Prenylnaringenin

8-prenylnaringenin is a prenylflavonoid, more specifically, a xanthohumol metabolite, found in the Citrus genus of plants belonging to the Rutaceae family [24] and exclusively available in nominal concentrations in citrus fruits such as oranges, lemons, grapefruits, and tangerines. Prenylflavonoids are a sub-class of the flavonoid group that represents a group of secondary metabolites derived from 2-phenylchromen-4-one and present a prenyl group attached to the flavone nucleus. Findings from previous studies reported that 8-prenylnaringenin had a protective effect on menopausal and post-menopausal symptoms, as well as exhibiting anticancer activities by the induction of autophagy or by modulating the cell cycle and suppressing the growth of tumor cells when tested in different types of in vitro experimental model systems [25]. It reduced oxidative stress, inflammatory processes, and the secretions of angiogenic factors. It also acted on vascularization processes, such as angiogenesis [26]. 

In an animal model (C57Bl/6 mice) of type 2 diabetes mellitus induced by a high-fat diet (HFD), Luís et al. showed that 8-prenylnaringenin normalized the expression of Galectin-3 (Gal3), a protein overexpressed during the diabetic state, and was strongly associated with oxidative stress in the liver and kidneys of diabetic mice. In addition, it reduced 3-nitrotyrosine (a marker of cell damage), inflammation, and nitric oxide (NO) production, and promoted the production of glycation end products (AGEs) [27]. Increased levels of AGEs in response to diabetic inflammation have been reported to play a role in tissue stiffness, increased blood pressure, heart failure, and endothelial dysfunction [28]. This polyphenol may, therefore, be a potential therapeutic agent against diabetes mellitus.

### 2.2. Cosmosiin

Cosmosiin, also known as apigetrin or apigenin-7-O-glucoside, is a glycosyloxyflavone with the molecular formula of C_21_H_20_O_10_, that is, apigenin substituted by a beta-D-glucopyranosyl moiety at position 7 via a glycosidic linkage. It is found in a variety of citrus plant species, such as *Citrus grandis* (L.) Osbeck (*red wendun*) and *Citrus aurantium* Linn. of the family Rutaceae. Recently, cosmosiin and its derivatives have been suggested as diabetic therapies [29]. The antidiabetic effect of cosmosiin was reported by Rao et al. in their in vitro study using 3T3-L1 adipocyte cells. Cosmosiin exerted its protective effects through promoting adiponectin secretion, resulting in increased phosphorylation of the insulin receptor-β (IR-β). In addition, it had a positive effect on glucose transporter 4 (GLUT4) translocation [30]. Therefore, these results suggest that cosmosiin has insulin-like activity, which plays a vital role in stimulating glucose uptake into muscles and adipocytes, suggesting that this flavonoid could be beneficial for the management of type 2 diabetes mellitus and related complications.

### 2.3. Diosmin

Diosmin (3′,5,7-trihydroxy-4′-methoxyflavone-7-ramnoglucoside) is a flavone found in citrus fruits and the leaves of oranges and lemons. This flavone has some important biological activities, such as antioxidant, anti-inflammatory, and anti-apoptotic effects [31]. Diosmin was isolated for the first time in 1925 from *Scrophularia nodosa* Linn. (a perennial herbaceous plant from the family Scrophulariaceae) and used for the first time in 1969 as a therapeutic agent for inflammatory disorders. Currently, it is a medication mainly used for the treatment of diseases, such as chronic venous insufficiency and hemorrhoids [32]. The effect of diosmin on lipid metabolism was evaluated using an animal model of streptozotocin (STZ)-induced diabetes [33]. Interestingly, it was shown to attenuate biochemical markers, such as fasting plasma glucose concentrations, glycosylated hemoglobin (HbA1c), and C-reactive protein (CRP). In addition, it decreased the levels of plasma lipids, including triglycerides (TG), free fatty acids, phospholipids, low-density lipoprotein cholesterol (LDL-C), and very low-density lipoprotein cholesterol (VLDL-C), and decreased high-density lipoprotein cholesterol (HDL-C). Besides, the activity of 3-hydroxy-3-methyl-glutaril-CoA reductase (HMG-CoA reductase), an important enzyme of the metabolic pathway that produces cholesterol, was enhanced in the liver and kidneys of diabetic rats but was inhibited by diosmin treatment. Finally, the activities of the lipoprotein lipase (LPL) and lecithin cholesterol acyl transferase (LCAT) enzymes were also altered by diabetes and normalized by diosmin.

Jain, Bansal, Dalvi, Upganlawar, and Somani [34] showed protective effects of diosmin against biochemical, behavioral, and oxidative stress parameters related to diabetic neuropathy in type 2 diabetic rats fed with an HFD. Diosmin also increased the threshold of nociception in thermal hyperalgesia and tail-flick tests, and improved motor capacity in diabetic rats. In addition, this flavonoid demonstrated a protective effect against oxidative stress, reducing markers of lipid peroxidation (malondialdehyde (MDA) and NO) levels. It also increased the activity of antioxidant enzymes, such as superoxide dismutase (SOD) and reduced glutathione (GSH), suggesting that it may help prevent the early development of diabetic neuropathy in rats.

Hsu, Lin, Cheng, and Wu [35] concluded that diosmin has a beneficial effect through the activation of the imidazoline I-2 receptor (I-2R) and opioid secretion. Diosmin induced β-endorphin-like immunoreactivity secretion in isolated adrenal glands in vitro via calcium-dependent reactions, which evidenced its utility as an antidiabetic drug via inducing opioid secretion. In addition, diosmin attenuated increased plasma glucose concentrations and increased hepatic glycogen levels in diabetic rats. It also activated the I-2R to promote metabolic homeostasis, resulting in reduced blood glucose and lipids in diabetic rats. It is worth mentioning that the administration of diosmin did not produce changes in body weight, food intake, or plasma insulin levels. 

Furthermore, diosmin has been reported to have therapeutic potential for behavioral parameters, such as the antinociceptive response and locomotor activity, as well as for the regulation of nociceptive biomarkers linked to the neuropathy caused by diabetes [34]. Taken together, it is suggested that diosmin can attenuate primary effects of diabetes, such as disturbances in plasma glucose and lipoproteins, by modulating key enzymes that regulate glucose metabolism and antioxidant activity.

### 2.4. Nobiletin

Nobiletin, chemically known as 5,6,7,8,3′,4′-hexamethoxyflavone, is a dietary flavone with the empirical formula C_21_H_22_O_8_ and the molecular weight 402.39. It is found in the peel of various citrus fruits, such as oranges and tangerines [36]. Like other bioflavonoids, nobiletin has shown potential medicinal properties in several pathologies and their associated causes, such as preventing type 2 diabetes [37], protecting against bone mineral density loss [38], treating cancer [39], and lowering blood cholesterol [40]. Studies have suggested that this flavone may be an effective therapeutic molecule for the treatment of metabolic syndromes, such as cardiovascular disease, abdominal obesity, and increased blood pressure.

Mulvihill et al. [41] evaluated its effect on lipoprotein secretion in cultured human hepatoma cells (HepG2) and a mouse model of dyslipidemia and atherosclerosis accompanying insulin resistance. The in vitro results showed that the administration of nobiletin dose-dependently reduced the secretion of apolipoprotein B100-containing lipoproteins, which represents an important risk factor for cardiovascular diseases. This effect happened through the activation of a mitogen-activated protein kinase/extracellular signal-regulated kinase (MAPK/ERK) pathway. MAPK/ERK activation by nobiletin decreased the mRNA expression of microsomal triglyceride transfer protein (MTP), diacylglycerol O-acyltransferase (DGAT)-1, and DGAT2, while it increased hepatic low-density lipoprotein (LDL) receptor (LDLR) mRNA expression. However, the authors found no evidence of nobiletin modulating the tyrosine phosphorylation of the insulin receptor (IR) or the insulin receptor substrate 1 (IRS-1). In addition, TG synthesis and TG mass were significantly lowered in nobiletin-treated cells, with the increase in the mRNA expression of carnitine palmitoyltransferase I α (CPT1-α) and peroxisome proliferator-activated receptor gamma coactivator 1-alpha (PGC1-α). Most of the favorable results observed in the abovementioned in vitro experiment were comparable with the in vivo results, in which nobiletin prevented diet-induced weight gain and reduced dyslipidemia in HFD-fed diabetic mice. The in vivo studies on nobiletin significantly decreased plasma lipids levels (TG and total cholesterol (TC)), reduced the very low-density lipoprotein-total triglyceride (VLDL-TG) secretion rate, and normalized elevated plasma non-esterified fatty acid (NEFA) and glycerol, in addition to reducing TG in both the liver and intestines in HFD-fed obese diabetic mice. In contrast with the in vitro results, nobiletin did not affect the hepatic expression of MTP or DGAT1/2. Nobiletin-treated HFD-fed obese diabetic mice also increased the expression of CPT1-α and PGC1-α, and the rates of fatty acid oxidation. Glucose tolerance tests conducted in the HFD-fed obese diabetic mice revealed that nobiletin normalized the impaired high-fat-diet-induced glucose tolerance, while significantly diminishing hyperinsulinemia and improving insulin sensitivity. Moreover, diet-induced obesity and adipocyte hypertrophy were inhibited in nobiletin-treated HFD-fed obese diabetic mice. It prevented dyslipidemia and hepatic steatosis, and improved metabolic parameters, leading to the prevention of atherosclerosis and a dramatic reduction in the lesions within the aortic sinus compared with the high-fat-diet-fed mice that were not treated with nobiletin.

Onda, Horike, Suzuki, and Hirano [42] added some important evidence from an in vitro study, which showed that nobiletin affected glucose uptake in insulin target cells such as adipocytes, using adipocyte cell lines (murine 3T3-F442 preadipocytes). Nobiletin treatment increased the uptake of [3H]-deoxyglucose in differentiated adipocytes in the presence of insulin. The influence on increased glucose uptake in the adipocytes was associated with several signaling cascade inhibitors that are recognized to promote pathways for glucose incorporation. The results showed that the phosphoinositide 3-kinases (PI3K), protein kinase B (Akt), and the protein kinase A (PKA) pathways were involved in the increase in glucose uptake. These in vitro results encourage further in vivo studies to analyze the antidiabetic action of this polymethoxyflavonoid and its molecular mechanism involved in enhancing glucose uptake via the PI3K/Akt signaling pathway in the insulin target tissues. 

To elucidate the antidiabetic mechanism of nobiletin in adipocytes, Kanda et al. [43] conducted a study using 3T3-L1 preadipocyte cells in which nobiletin suppressed lipid accumulation in 3T3-L1 adipocytes, suggesting that nobiletin inhibited adipogenesis in 3T3-L1 cells when the adipocyte differentiation was induced by insulin, 3-isobutyl-1-methylxanthine (IBMX), and dexamethasone (DEX). Regarding the mechanism of action involved in this response, nobiletin did not affect the protein expression of peroxisome proliferator-activated receptor gamma (PPARγ)1 in 3T3-L1 cells; however, it significantly suppressed PPARγ2 protein expression, an important marker in adipogenesis. The transcripts of PPARγ2 and adipocyte protein 2 (aP2), two target genes of PPARγ, were significantly down-regulated by nobiletin treatment. In addition, it suppressed CCAAT-enhancer-binding protein beta (C/EBPβ) expression, suggesting that nobiletin may inhibit adipocyte differentiation by down-regulating PPARγ2 gene expression via decreasing C/EBPβ expression. Finally, nobiletin reduced the phosphorylation of the cAMP-response element-binding protein (CREB) and strongly improved the phosphorylation of the signal transducer and activator of transcription 5 (STAT5), suggesting that a suppressive effect of nobiletin on adipocyte differentiation was involved due to the enhanced activation of STAT5 by the regulation of PPARγ activity.

Furthermore, Parkar, Bhatt, and Addepalli [44] hypothesized that nobiletin, due to its metalloproteinase (MMP)-2- and MMP-9-inhibitory and antioxidant potential, could ameliorate the cardiovascular dysfunction in diabetes. In an animal model of STZ-induced diabetes, nobiletin treatment reduced the mean arterial pressure in the diabetic rats in comparison to vehicle-treated rats. Heart rate fell rapidly and dramatically after the administration of STZ; however, nobiletin increased the heart rate and kept the condition normal. Additionally, in connection to other cardiac parameters, nobiletin reduced MMP-2 and MMP-9 enzymatic activities in the heart, improved the cardiac hypertrophy index, attenuated the deterioration in the morphology of cardiomyocytes, and reduced diabetes-induced myocardial fibrosis in the rats. Nobiletin also showed antioxidant effects by improving myocardial SOD and catalase activity and decreasing MDA levels. Moreover, nobiletin ameliorated vascular reactivity and collagen levels in the aortae of rats. 

Zhang et al. [45] also investigated the therapeutic effect of nobiletin on STZ-induced diabetic cardiomyopathy in mice. Echocardiography and hemodynamic measurements showed a protective effect on cardiac function in nobiletin-treated mice. Nobiletin treatment significantly attenuated the mRNA expression of nicotinamide-adenine dinucleotide phosphate-reduced (NADPH) oxidase isoforms (p67phox, p22phox, and p91phox), suggesting its potential antioxidant effect. Nobiletin improved SOD1 activity and decreased MDA levels in cardiac tissue, reinforcing the positive influence on oxidative-stress-related disorders. These effects were also accompanied by anti-inflammatory responses, such as the modulation of the mRNA expression of pro-inflammatory cytokines (tumor necrosis factor-alpha (TNF-α) and interleukin (IL)-6) in diabetic myocardium treated with nobiletin. In addition, the authors highlighted the fact that nobiletin treatment did not produce any significant effect on blood glucose levels. However, the treatment decreased the expression of transforming growth factor (TGF)-β1, connective tissue growth factor (CTGF), fibronectin, and collagen Iα, and also reduced cardiac fibrosis in the nobiletin-treated mice. Nobiletin also reduced phosphor-kappaB-α (IκB-α) expression, with the subsequent inhibition of phosphor-p65 activity. These results indicate that the treatment with nobiletin mitigated cardiac dysfunction and interstitial fibrosis, which may be due to its constructive action on the suppression of the c-Jun N-terminal kinase (JNK), P38, and nuclear factor kappa B (NF-κB) signaling pathways.

A recent study tested the hypothesis that nobiletin provides metabolic protection against the phosphorylation of AMP-activated protein kinase (AMPK) and acetyl-CoA carboxylase (ACC) in three different mouse models; mice deficient in hepatic AMPK (*Ampkβ1-/-*), mice incapable of the inhibitory phosphorylation of ACC (*AccDKI*), and mice with adipocyte-specific AMPK deficiency (*iβ1β2AKO*) [46]. Nobiletin was able to activate (increase the phosphorylation of) AMPK in human hepatocarcinoma HepG2 cells in the presence of high glucose. Additionally, ACC phosphorylation, which was suppressed by hyperglycemia, was reversed through nobiletin treatment. In vitro nobiletin-treated cells had reduced lipogenesis and increased fatty acid oxidation independent of AMPK. In summary, the results of this study showed that nobiletin treatment attenuated obesity, hepatic steatosis, dyslipidemia, and insulin resistance, and protected metabolism in three mouse models independently of AMPK activation. The authors also emphasized the potential therapeutic convenience of this citrus flavonoid nobiletin, specifically in the management of metabolic syndromes such as diabetes and obesity, and further in-depth studies are warranted to investigate the primary mechanism of action that influences insulin sensitivity.

Nobiletin has, therefore, been shown to have potent antidiabetic, anti-obesity, and hypolipidemic effects by modulating several physiological pathways. In addition, it acted as an immunomodulatory molecule, attenuating inflammatory and oxidative stress markers, which are linked to the various diabetic complications. This evidence reinforces the therapeutic potential of nobiletin for diabetes in in vitro experimental systems and animal models, which should be further verified in humans.

### 2.5. Rhoifolin

Rhoifolin, with the molecular formula C_27_H_30_O_14_, is one of the most common flavonoids and is used extensively in preclinical investigations to explore its pharmacological effects in a wide range of chronic diseases including diabetes and obesity. It is found in several citrus fruits such as bitter oranges, grapefruits, lemons, and unripe grapes [47,48]. Previous studies found that rhoifolin influenced several biological activities, with antioxidant [49], anti-inflammatory [50], hepatoprotective [51], and anticancer potential [52].

Rhoifolin was also isolated from *Citrus grandis* (L.) Osbeck leaves, and its insulin-mimetic action was reported by Rao et al. in an in vitro study using differentiated 3T3-L1 adipocytes cells. It was found to act via promoting adiponectin secretion, the phosphorylation of IR-β, and GLUT4 translocation, which are considered to be critically involved in diabetic complications [30]. The action of rhoifolin against these genes may provide novel targets for combating insulin-resistance-associated diseases.

**Table 1:** The main characteristics of in vitro studies using citrus flavonoids for the management of diabetes mellitus.

Flavonoids	Class	Concentrations and Duration of the Treatment	In Vitro Models	Effects and Molecular Mechanisms	Ref.
Nobiletin	Flavone	1, 2.5, 5, 10, and 20 µM; 24 h	HepG2 cells (human hepatoma cells)	Nobiletin activated mitogen-activated protein kinase-extracellular signal-related kinase (MAPK/ERK), resulting in the marked inhibition of apolipoprotein B100 secretion. It neither induced the phosphorylation of the insulin receptor (IR) or insulin receptor substrate-1(IRS-1) tyrosine nor triggered lipogenesis associated with insulin resistance.	[41]
Rhoifolin and cosmosiin from *Citrus grandis* (L.) Osbeck leaves	Flavone	Rhoifolin: 0.001–5 μM; cosmosiin: 1–20 μM; 24 h	3T3-L1 adipocyte cells	Rhoifolin and cosmosiin exerted antidiabetic effects by promoting adiponectin secretion, the tyrosine phosphorylation of IR-β, and glucose transporter 4 (GLUT4) translocation. These bioactive molecules may help in insulin resistance-related treatment for diabetic complications.	[30]
Tangeretin and nobiletin	Flavone	5–50 mM; 24 h	3T3-F442A preadipocytes	Tangeretin and nobiletin induced increased glucose uptake in murine adipocytes, suggesting that the action was mediated by phosphatidylinositol 3-kinase (PI3K) as well as protein kinase B (Akt) and protein kinase A (PKA)/cAMP-response element-binding protein (CREB) signaling-dependent pathways.	[42]
Flavonoids from *Citrus aurantium* Linn. include naringin, esperidin, poncirin, sosiennsetin, sineesytin, nobiletin, and tangeretin	Flavone and flavanone	0, 10, and 50 μg/mL; 0–6 days	3T3-L1 preadipocytes	*C. aurantium* containing flavonoids decreased the expression of key adipocyte differentiation regulators, including CCAAT-enhancer binding protein family (C/EBPβ and C/EBPα) and peroxisome proliferator-activated receptor gamma (PPARγ); it reduced adipogenesis and the accumulation of cytoplasmic lipid droplets during differentiation in 3T3-L1 cells.	[53]
Nobiletin	Flavone	0, 1, 10, and 100 μM; 7 days	3T3-L1 preadipocytes	Nobiletin suppressed the differentiation of 3T3-L1 preadipocytes into adipocytes by down-regulating the expression of the gene coding for PPARγ2. In addition, nobiletin reduced the phosphorylation of CREB and strongly improved the phosphorylation of signal transducer and activation of transcription (STAT)5.	[43]
Sudachitin	Flavone	30 mmol/L; 48 h	Primary myoblasts	Sudachitin increased mitochondrial biogenesis and improved mitochondrial function, leading to an improvement in lipid and glucose metabolism mediated via the sirtuin (Sirt) 1-AMP-activated protein kinase (AMPK)-peroxisome proliferator-activated receptor gamma coactivator-1- alpha (PGC-1α) pathway.	[54]
Naringenin	Flavanone	0, 10, and 50 μM; 3 h	RAW 264 (macrophages) cells and 3T3-L1 adipocytes	Naringenin inhibited the monocyte chemoattractant protein-1 (MCP-1)’s mRNA expression and secretion in the adipocytes in a dose-dependent manner. It also prevented the MCP-1 production stimulated by the interaction between the adipocytes and the infiltrated macrophages.	[55]
Hesperidin and naringin	Flavanone	0.25, 0.5, 1, and 2 mg/mL; 1 and 24 h	Pancreatic islets	Hesperidin and naringin increased the production and the release of insulin from the islet cells and decreased the intestinal glucose absorption.	[56]
Quercetin	Flavanol	10 and 100 mM; 24 h	L6 myotubes	Quercetin activated the adenosine monophosphate kinase (AMPK)-P38 MAPK pathway and up-regulated glucose transporter type 4 (GLUT4)/AKT mRNA expression to induce glucose uptake in skeletal muscle cell lines.	[57]
Diosmin	Flavone	0.01–1 μmol/L; 24 h	Transfected imidazoline receptor (I-R) gene in CHO-K1 cells (Chinese hamster ovary cell)	Diosmin enhanced calcium influx in I-R gene-transfected CHO-K1 cells. Diosmin effectively activated the I-R gene via inducing opioid secretion, showing utility as an antidiabetic drug.	[35]
Hesperidin	Flavanone	12.5, 25, and 50 μmol/L; 6 h	RGC-5 cells (retinal ganglial cells)	Hesperidin protected against a high level of glucose-induced cell apoptosis by down-regulating caspase-9, caspase-3, and Bax/Bcl-2. Furthermore, it significantly inhibited the phosphorylation of c-Jun N-terminal kinases (JNK) and activated p38 MAPK in high glucose-fed RGC-5 cells.	[58]
Hesperidin and hesperetin	Flavanone	40, 80, 120, 160, and 200 μM; 24 h	Rat liver cells	Flavonoids hesperidin and hesperetin inhibited the activities of two gluconeogenesis enzymes, alanine aminotransferase (ALT) and aspartate aminotransferase (AST), indicating their effectiveness in treating AST and ALT-mediated metabolic disorders, including in diabetes mellitus.	[59]
Tangeretin	Flavone	0, 2.5, 5, and 10 μM; 24 h	Human glomerular mesangial cells (MCs)	Tangeretin very effectively inhibited high glucose (HG)-induced cell proliferation, oxidative stress, and extracellular matrix (ECM) expression in the human glomerular mesangial cells (MCs) via inactivating the extracellular signal-regulated kinase (ERK) signaling pathway. It also displayed therapeutic potential in the management of diabetic nephropathy.	[60]
Didymin	Flavanone	10 and 20 μM; 6 and 24 h	Human umbilical vein endothelial cells (HUVECs)	Didymin protected against high glucose (HG)-induced human umbilical vein endothelial cells by modulating the expression of intercellular adhesion molecule (ICAM)-1 and vascular cell adhesion protein (VCAM)-1, and regulating nuclear factor kappa B (NF-κB)-mediated inflammatory cytokines and chemokines. Didymin prevented HG-induced endothelial dysfunction and death via antioxidative and anti-inflammatory activities.	[61]
Didymin	Flavanone	10–30 μM; 15 and 30 min, 1 and 24 h, 28 days	HepG2 (human hepatocarcinoma) cell line	Didymin inhibited α-glucosidase, activated the insulin-signaling pathway, and improved insulin sensitivity. It showed potent inhibitory activity against the key enzymes involved in diabetes mellitus, including protein tyrosine phosphatase 1B (PTP1B), α- glucosidase, advanced glycation end products (AGEs), and aldose reductase (AR).	[62]
Naringenin	Flavanone	0.01–1 μM; 1 and 24 h	NSC34 (mouse neuroblastoma and embryonic spinal cord motor neurons) cell line	Naringenin suppressed neuronal apoptosis and enhanced antioxidant protective effects in methylglyoxal (MG)-treated NSC34 cells. It prevented MG-induced hyperglycemia-related neurotoxicity via regulating insulin-like growth factor 1 receptor (IGF-1R)-mediated signaling.	[63]
Naringenin	Flavanone	0, 1, 10, and 50 μM; 30 min, 3 and 6 h	3T3-L1 (adipocytes) and RAW264 (macrophages) cells	Naringenin inhibited monocyte chemotactic protein (MCP)-3 expression in 3T3-L1 adipocytes and a coculture of 3T3-L1 adipocytes and RAW264 macrophages. It did not affect the expression of macrophage inflammatory protein-2 (MIP-2), a key chemokine for neutrophil migration and activation, in macrophages or a coculture of adipocytes and macrophages.	[64]
Nobiletin	Flavone	10 μM; 1 and 4 h	HepG2 (human hepatocarcinoma) cell line	Nobiletin increased pAMPK in HepG2 incubated with high glucose content, in which the phosphorylation of AMPK was suppressed, which was comparable to the action carried out by the reference standards (resveratrol and metformin).	[46]

### 2.6. Sudachitin

Sudachitin (5,7,4′-trihydroxy-6,8,3′-trimethoxyflavone), also known as menthocubanone, is a polymethoxylated flavone originally found in the skin of *Citrus sudachi* Hort. fruit. Sudachitin belongs to the class of organic compounds known as 8-O-methylated flavonoids and has been detected commonly in citrus fruits, such as mandarin oranges and bitter oranges [65]. It exhibits diverse biological activities, such as the suppression of inflammatory bone destruction [66], induction of apoptosis in human keratinocyte HaCaT cells [67], enhancement of antigen-specific cellular and humoral immune responses [68], and inhibition of matrix metalloproteinase (MMP)-1 and MMP-3 production in TNF-α-stimulated human periodontal ligament cells [69].

The effects of sudachitin on glucose, lipid, and energy metabolism in an HFD experimental obesity model using C57BL/6J mice and diabetic *db/db* mice fed a normal diet were investigated by Tsutsumi et al. [54], and it was found that sudachitin reduced the weight gain in the HFD mice without changing the food intake. It also ameliorated the elevated adipose tissue mass, increased subcutaneous fat deposits, and elevated visceral fat composition, and normalized adipocyte size and function. In addition, it reduced hyperinsulinemia and hyperglycemia, improved glucose tolerance, ameliorated plasma leptin levels, decreased visceral fat content, increased plasma adiponectin levels, and improved insulin sensitivity. A possible explanation for these effects could be the ability of sudachitin to modulate metabolism-related genes, such as by modulating the mRNA expression of GLUT4 and transcripts of uncoupling protein 1 and 3 (UCP1 and UCP3) in white adipose tissue and the liver, which were significantly increased in the white adipose tissue of the diabetic animals. Besides, it was able to decrease the levels of the mRNA transcripts encoding FAS ligand, ACC1, and ACC2 in the liver. Tsutsumi et al. also reported that sudachitin promoted energy expenditure by activating the sirtuin (Sirt)1–PGC1α pathway, increased basal muscle skeletal adenosine triphosphate (ATP) contents, and increased mitochondrial citrate synthase activity. Sudachitin also improved insulin sensitivity and reduced fasting blood glucose and TG levels in diabetic *db/db* mice. Finally, an important in vitro study carried out by Tsutsumi et al. showed that sudachitin influenced the mitochondrial biogenesis by activating vital signaling pathways in myocytes, increasing the expression of genes such as nuclear respiratory factor 1 and 2 (NRF1 and NRF2) and mitochondrial transcription factor A (mtTFA). Sudachitin treatment increased the mitochondrial number and activity. Therefore, these observations indicate that sudachitin has good potential for managing obesity and diabetes and its associated complications.

### 2.7. Tangeretin

Tangeretin is an O-polymethoxylated flavone with methoxy groups at positions 5, 6, 7, 8, and 4’, and is found in tangerines and orange peel. It performs a number of biologically beneficial activities and has antioxidant, anti-inflammatory, antitumor, hepatoprotective, and neuroprotective potential [70]. The properties of this flavonoid with respect to diabetes and its associated comorbidities have also been widely studied. Regarding the antidiabetic effects of tangeretin, in vitro evidence confirmed that it increased glucose uptake in differentiated 3T3-F442 adipocytes, even in the presence of insulin. In addition, results showed that 3T3-F442 adipocyte glucose uptake by the PI3K, Akt, and PKA pathways was increased following treatment with this polymethoxyflavonoid [42].

Sundaram, Shanthi, and Sachdanandam [71] evaluated the antihyperglycemic potential of tangeretin regarding the activities of key enzymes linked with carbohydrate and glycogen metabolism in diabetic rats. Tangeretin treatment reduced blood glucose to near-normal levels, increased hemoglobin (Hb), and decreased hemoglobin (Hb)A1c levels, besides reversing the obese body weight and liver weight changes induced by diabetes. In addition, tangeretin normalized the activities of key hepatic enzymes and reinstated the levels of glycogen and the activities of glycogen synthase and glycogen phosphorylase. Histopathological analysis showed a significant increase in the regeneration of pancreatic β-cells in the islets of Langerhans in tangeretin-treated diabetic rats compared with those in the non-treated diabetic animals. 

Furthermore, Sundaram, Shanthi, and Sachdanandam [72] used a diabetic animal model to gain more therapeutic information on the mechanism of the action regarding the antioxidant, anti-inflammatory, and cardio-protective effectiveness of tangeretin. The oral administration of tangeretin reversed the body weight and heart weight changes by its insulinotropic action. Tangeretin administrated to the diabetic rats attenuated and normalized the lipid profiles in the plasma and cardiac tissues. These effects were mediated through the modification of the activities of key enzymes (LCAT, LPL, and HMG-CoA reductase) of lipid metabolism in the liver and increased GLUT4 expression in the heart tissues of diabetic rats. Moreover, tangeretin administration in diabetic rats decreased the levels of lipid peroxidation by increasing the activities of antioxidant enzymes (SOD, catalase (CAT), glutathione peroxidase (GPx), and glutathione reductase (GR)). It also caused a significant reduction in both inflammatory cytokines (TNF-α and IL-6) and cardiac marker enzymes (aspartate aminotransferase (AST), lactate dehydrogenase (LDH), and creatine phosphokinase (CPK)) in the plasma and heart tissues. Additionally, tangeretin treatment markedly decreased the nuclear translocation of NF-κB and TNF-α according to immunostaining in cardiac tissues. In summary, the results suggest that tangeretin treatment plays a beneficial role in regulating diabetes and its associated cardiovascular risk. 

Chen, Ma, Sun, and Zhu [60] elucidated the effects of tangeretin on high glucose-induced oxidative stress and extracellular matrix (ECM) accumulation in human glomerular mesangial cells (HGMCs) and discovered the underlying mechanisms. The important inflammatory factor TGF-β1’s expression induced by high glucose was efficiently suppressed in tangeretin-treated cells. The citrus molecule suppressed reactive oxygen species (ROS) and MDA production, while it increased SOD activity. In addition, high glucose treatment greatly increased the expression of fibronectin and collagen IV in HGMCs, which was then reversed by tangeretin treatment. The extracellular signal-regulated kinase (ERK) pathway plays an important role in the development of diabetic nephropathy, and this study concluded that tangeretin can modulate ERK signaling through preventing the activation of the ERK signaling pathway in high glucose-stimulated mast cells (MCs). These results highlighted tangeretin as a curative agent in the management of diabetic nephropathy, the leading cause of morbidity and mortality resulting in end-stage renal disease. 

Therefore, tangeretin has a promising role in research into diabetic therapy, since its effects appear to be consistent and reliable in diabetic preclinical studies. Its major effects include attenuating biochemical parameters related to diabetic conditions, modulating key enzymes of lipid and glycolytic metabolism, attenuating inflammation and oxidative-stress-signaling markers, and exhibiting protective effects on the heart and liver tissues, which are considered to be vital in diabetic metabolic disorders.

### 2.8. Didymin

Didymin ((S)-5,7-dihydroxy-4′-methoxyflavanone-7-β-rutinoside) is an oral bioactive citrus flavonoid-O-glycoside belonging to a flavanone class found in several citrus fruits, such as oranges, lemons, grapefruits, and mandarins [73]. Although it has great antioxidant potential, didymin is mainly mentioned in the literature in relation to its potent anticancer capacity, having an antiproliferative effect and preventing the growth of cancer cells [74,75,76]. The effects of didymin against endothelial dysfunction, a pathological process involved in atherogenesis, were described by Shukla, Sonowal, Saxena, and Ramana [61], who demonstrated the role of this flavanone in inhibiting the apoptosis of human umbilical vein endothelial cells induced by high glucose, via modulating oxidative stress signals, leading to the generation of ROS as well as the activation of caspase-3 and Erk1/2 and regulation of the Bcl2 protein. Moreover, didymin also alleviated high glucose-induced endothelial dysfunction by preventing monocyte adhesion to endothelial cells, restoring endothelial nitric oxide synthase (eNOS) and NO levels, reducing the levels of several inflammatory cytokines, such as TNF-α, interferon gamma (INF-γ), IL-1β, IL-2, and IL-6. Thus, these results demonstrated that it could be developed as a natural therapeutic agent against hyperglycemia-induced endothelial dysfunction and mortality.

Ali et al. [62] evaluated the antidiabetic potential of didymin and the molecular mechanisms underlying its effects in the insulin-resistant HepG2 cell line. In vitro experiments showed that didymin inhibited human recombinant aldose reductase (HRAR), rat lens aldose reductase (RLAR), α-glucosidase, and AGE formation. It also activated the insulin-signaling pathway and resulted in improved insulin sensitivity. Together, these physiological effects led to a potent antidiabetic effect. Regarding the molecular mechanisms related to these effects, didymin reduced the expression of protein tyrosine phosphatase (PTP1B) and increased the phosphorylation of IRS-1, PI3K, glycogen synthase kinase 3β (GSK3β), and Akt, besides reducing two key enzymes, leading to diminished hepatic glucose production in insulin-resistant HepG2 cells. Molecular docking studies indicated that didymin possessed high affinity and tight binding capacity for the active sites of HRAR, RLAR, PTP1B, and α-glucosidase. Additionally, didymin showed important vascular effects through the activation of molecular pathways that result in glycemic control, highlighting the great therapeutic potential for diabetes and diabetes-associated complications. Thus, further clinical trials are warranted to investigate the use of didymin as a potential lead candidate to protect against metabolic disorders targeting various organs.

### 2.9. Hesperidin

Hesperidin is a flavanone glycoside commonly found in citrus fruits such as oranges, tangerines, lemons, and grapefruits, and is one of the most important non-essential nutrients for human beings [77]. Its name originated from the word “hesperidium”, which denotes fruits derived from citrus trees. The consumption of hesperidin appears to influence blood pressure and improve antioxidant status in humans [78]. This citrus flavanone is a widely used dietary supplement, alone or in combination with other bioflavonoids, for the treatment or prevention of disturbances in the vascular system (reducing capillary permeability) and as an anti-inflammatory, antioxidant, or anticarcinogenic herbal medicine [79,80]. The majority of the medicinal properties of this bioflavonoid have been attributed to its ability to modulate pro-inflammatory cytokines, such as TNF-α, IL-1β, and IL-6, and reduce inflammation and oxidative stress in biological systems, as demonstrated in different animal models of inflammatory reactions [81].

One of the most common chronic diseases that can be treated with this flavonoid is diabetes, as Akiyama et al. [82] reported. The authors used an animal model of STZ-induced diabetes to assess the effect of hesperidin on biochemical markers, glucose-regulating enzymes, and parameters of bone loss in marginal type 1 diabetic rats. Hesperidin reduced blood glucose and serum insulin and normalized the enzymatic activities of glucose-6-phosphatase (G6Pase), glucokinase (GK), and other hepatic enzymes important in glycemic control. In addition, Mahmoud et al. [83] showed that hesperidin treatment could attenuate hyperglycemia-mediated oxidative stress and suppress the production of pro-inflammatory cytokines, such as TNF-α and IL-6, in HFD/STZ-induced type 2 diabetic rats. These results corroborate those of El-Marasy et al. [84], who reported that the oral administration of hesperidin reduced blood glucose, decreased levels of MDA and IL-6, and increased GSH and brain-derived neurotropic factor (BDNF) levels in the brains of rats with diabetes. Hesperidin also normalized the levels of monoamines in the brain, specifically, norepinephrine and dopamine, and elevated brain levels of serotonin. The results obtained were also reflected in physical and behavioral parameters, since hesperidin reduced the immobility time of rats with diabetes in the forced swimming test.

Visnagri, Kandhare, Chakravarty, Ghosh, and Bodhankar [85] evaluated the effect of hesperidin against diabetic neuropathic pain in rats. Hesperidin treatment inhibited the reduction in motor nerve and sensory nerve conduction velocity induced by diabetes. Impaired neural conduction velocity can affect sensory, nociceptive, and motor responses. However, hesperidin normalized the sensorial responses by attenuating the increased mechanical and thermal hyperalgesia in diabetic rats. This bioflavonoid also attenuated several diabetic biochemical parameters, such as high blood glucose, TC, and serum TG, increased the plasma concentration of insulin, and had positive effects on hemodynamic variables, important in the treatment of diabetes and the associated cardiovascular complications. Finally, hesperidin showed neural-protective effects, accompanied by a reduced infiltration of neutrophils and macrophages in the sciatic nerve and reduced mRNA expression of neural TNF-α and IL-1β, two important pro-inflammatory cytokines involved in the progression of diabetes. In addition, it restored the distortion of the architecture of the sciatic nerve caused by STZ-induced necrosis, edema, and congestion on nerve fibers. 

Mahmoud, Ahmed, Ashour, and Abdel-Moneim [56] generated more information regarding the mechanism of action of hesperidin as a natural antidiabetic product using HFD/STZ-induced type 2 diabetic rats and in vitro studies. The oral administration of hesperidin was shown to reduce fasting glucose and attenuate insulin resistance in diabetic rats, and increase the release of insulin in isolated pancreatic islets. In addition, it normalized the activities of metabolic enzymes, such as glucose-6-phosphatase, glycogen phosphorylase, and fructose-1,6-bisphosphatase. This bioflavonoid was also found to increase glucose uptake in isolated pancreatic cells. It has been shown that the antidiabetic effects of hesperidin are mainly due to its capacity to increase the mRNA and protein expression of GLUT4 in adipose tissue, in addition to decreasing intestinal glucose absorption.

Liu, Liou, Hong, and Liu [58] conducted experiments to evaluate the effects and mechanisms of hesperidin on different pathophysiological parameters of diabetic retinopathy using retinal RGC-5 ganglial cells. It is well established that oxidative stress plays an important role in diabetes, and it is characterized by high concentrations of ROS and the lipid peroxidation marker MDA, as well as a reduction in the activity of antioxidant enzymes. The higher levels of intracellular ROS, MDA, and protein carbonyl in RGC-5 cells under high concentrations of glucose were down-regulated by hesperidin, and the reduced activities of SOD, GPx, and catalase (CAT) were recovered. The authors also showed that hesperidin blocked the high glucose-induced elevation of the cell apoptosis regulator Bax and decreased Bcl-2 concentrations in high glucose-exposed RGC-5 cells. It also down-regulated caspase-9 and caspase-3, lowered the Bax/Bcl-2 ratio, and restored mitochondrial function, confirming that cells can be protected by hesperidin from high glucose-induced apoptosis through a mitochondrially mediated pathway. Moreover, this citrus flavone significantly inhibited the phosphorylation of JNK and activated p38 mitogen-activated protein kinases (p38 MAPK), proving its vital effect of protecting cells from ROS injury and cellular death. 

According to Zareei, Boojar, and Amanlou [59], alanine aminotransferase (ALT) and AST are two liver pyridoxal phosphate-dependent enzymes involved in gluconeogenesis and amino acid metabolism that catalyze the intermediary reactions of glucose and protein metabolism. The increased activity of these enzymes has been observed in liver metabolic syndrome, atherogenesis, and type I and type II diabetes. In rat liver cells, different concentrations of hesperidin exhibited inhibitory effects against ALT and AST activities; therefore, it can be considered a potential compound for designing a safe and effective agent for the management of diabetes mellitus-associated hepatic injury.

Li, Kandhare, Mukherjee, and Bodhankar [86] conducted several experiments in diabetic animals aiming to evaluate the effectiveness of hesperidin against diabetic foot ulcers induced by injecting STZ followed by excision wounds created on the dorsal surface of the foot. In addition, hesperidin treatment in diabetes-induced rats inhibited weight loss, reduced insulin concentrations, normalized blood glucose, reduced food and water intake, increased SOD and GSH levels, and reduced MDA and NO levels. The results presented by Li, Kandhare, Mukherjee, and Bodhankar also showed that this flavanone exhibited beneficial effects in the treatment of wounds, since it attenuated the morphological changes caused, and reduced the edema and inflammatory infiltration of polymorphonuclear cells, in addition to accelerating angiogenesis and vasculogenesis. These effects can be attributed to the regulatory action of hesperidin on important biomarkers, such as vascular endothelial growth factor c (VEGF-c); angiopoietin-1, the ligand for the tyrosine kinase receptor Tie2 (Ang-1/Tie-2); TGF-β; and Smad-2/3. 

In STZ-induced diabetic animals, Dokumacioglu et al. [87] supported some of the previously reported data about this citrus flavonoid hesperidin and added more important molecular findings, which showed that hesperidin significantly decreased serum total cholesterol, TG, LDL C, VLDL C, and MDA levels and increased GSH concentrations but did not change HDL-C levels. Additionally, histological analysis showed that treatment with hesperidin led to an improvement in the degenerated islet cells in diabetic rats. The study also reported a reduction in pro-inflammatory cytokines in diabetic rats. The authors speculated that the control of weight loss in the diabetic rats treated with hesperidin might result from the organized regulation of TNF-α and IL-6 levels in adipose tissue. Studies reported that the increased secretion of TNF-α and IL-6 by subcutaneous fat tissue correlates with obesity and adiposity, and such was also suggested to be associated with the origination of diabetic microvascular complications [88,89].

### 2.10. Hesperetin

Hesperetin ((S) -2,3-dihydro-5,7-dihydroxy-2- (3-hydroxy-4-methoxyphenyl) -4-benzopyran), an important citrus flavonoid and aglycone form of hesperidin, is a bitter compound mainly found in bitter oranges and lemons [90]. It is interesting to note that hesperetin has a higher bioavailability compared to hesperidin due to the rutinoside moiety attached to the flavonoid, and this seems to contribute to its superior anti-inflammatory and antioxidant properties [91]. Hesperetin is widely studied in several pathological conditions and exhibits neuroprotective effects [92], anticancer properties [93], anti-neuroinflammatory potential [94], antioxidant effects [95], and anti-inflammatory [96] activities, among others. 

Zareei, Boojar, and Amanlou [59] investigated and evaluated the effect of hesperetin on the AST and ALT enzymes in the liver of rats and concluded that hesperetin exclusively inhibited ALT and AST activities in diabetes-induced rats. Therefore, their study hypothesized that hesperetin may be a potential compound for designing safe and effective drugs for the management of increased ALT- and AST-related disorders, which are especially found in diabetes. Furthermore, Revathy, Subramani, Sheik Abdullah, and Udaiyar [97] showed that hesperetin exhibited an antihyperglycemic effect by reducing blood glucose and enhancing plasma insulin and glycogen levels in an animal model of STZ-induced diabetes. Hesperetin treatment ameliorated vascular congestion and mononuclear cellular infiltration, and improved hepatic architecture, which was damaged by profound hyperglycemia. Hesperetin also alleviated the abnormality caused by hyperglycemia in pancreatic β-cells, inducing a notable extension of islets, improved staining in pancreatic β-cells, and boosting the number of insulin immune-positive cells of the islets. It also recovered the diabetes-induced damaged kidney tissue by reducing marked tubular necrosis, improving the architecture of the glomerulus and renal cortex, and attenuating interstitial inflammation in rat renal tissues.

Samie, Sedaghat, Baluchnejadmojarad, and Roghani [98] assessed the beneficial effect of hesperetin on diabetes-associated testicular injury in diabetic rats. Like other bioflavonoids, hesperetin was also able to prevent body weight loss, DNA fragmentation, and testicular oxidative stress and/or apoptosis; increase serum testosterone levels; reduce serum glucose, MDA, ROS, and protein carbonyl levels; and prevent caspase 3 activity in diabetic animals. Hesperetin treatment also showed important antioxidant effects by increasing glutathione, mitochondrial membrane potential (mMP), and ferric reducing antioxidant power (FRAP) levels, besides improving the activities of enzymes such as SOD, CAT, and GPx. Finally, hesperetin showed positive effects on testicular function and improved sperm counts, motility, and viability, as well as reducing inflammatory cytokines (TNF-α and IL-17) and preventing damage to the seminiferous tubules in diabetic rats. 

Overall, hesperetin exhibits anti-inflammatory and antioxidant effects in diabetes-mediated metabolic disorders. These results suggest that hesperetin specifically modulates biochemical parameters linked to liver enzymes, in addition to protecting the vital organs affected by the deleterious effects of profound hyperglycemia. Thus, further clinical trials should be carried out to verify hesperetin as an important potential treatment against diabetes and related metabolic complications.

**Table 2:** Description of the main characteristics of animal studies using citrus flavonoids for the management of diabetes mellitus.

Flavonoids	Class	Animal Models	Dose/Route/Duration of the Experiment	Effects and Molecular Mechanisms	Ref.
Hesperidin	Flavanone	Wistar Rats	Hesperidin-containing animal diet (10 g/kg diet); 28 days	Hesperidin attenuated hyperglycemia and hyperlipidemia by decreasing blood glucose and normalizing hepatic glucose-regulating enzyme activities but did not affect bone tissue and bone metabolic parameters in streptozotocin (STZ)-injected marginal diabetic weanling rats.	[82]
Rutin	Flavonol	Wistar Rats	50 mg/kg (*intraperitoneal*); 45 days	Rutin significantly reduced the blood glucose level, improved the lipid profiles, and normalized the activities of hepatic enzymes in STZ-induced diabetic rats. It also regulated hyperglycemia and dyslipidemia, and inhibited the progression of liver and heart dysfunction in STZ-induced diabetic rats.	[99]
Nobiletin	Flavone	C57BL/6 *Ldlr*-/- Mice	Nobiletin (0.1 or 0.3% mixed in high-fat Western diet); 56 to 182 days	Nobiletin regulated liver biomarkers by increasing hepatic and peripheral insulin sensitivity, improving glucose tolerance, and protecting against the development of atherosclerosis.	[41]
Naringenin	Flavanone	Wistar Rats	10 mg/kg (*intraperitoneal*); 35 days	Naringenin ameliorated aortic reactivity dysfunction in diabetic rats by attenuating lipid peroxidation and oxidative injury via a nitric acid-dependent pathway.	[100]
Hesperidin and naringin	Flavanone	Wistar Rats	50 mg/kg (*oral administration*); 28 and 30 days	Hesperidin and naringin lowered the level of pro-inflammatory cytokine (tumor necrosis factor-alpha (TNF-α) and interleukin (IL)-6) production and enhanced antioxidant defenses in a type 2 diabetes rat model by normalizing the altered blood glucose and antioxidant parameters in the liver.	[83]
Diosmin	Flavone	Wistar Rats	100 mg/kg (*intragastric*); 45 days	Diosmin attenuated lipid abnormalities in the diabetic rats via reducing the plasma and tissue lipids significantly, along with a profound increase in high-density lipoprotein cholesterol (HDL-C) levels.	[33]
Hesperidin	Flavanone	Wistar Rats	25, 50, or 100 mg/kg (*oral administration*); 21 days	Hesperidin reduced hyperglycaemia, decreased malondialdehyde (MDA) and IL-6 levels, and enhanced the brain-derived neurotrophic factor (BDNF) and monoamines in the brain, thereby enabling it to be effective in treating and managing neurogenesis in diabetic rats.	[84]
Naringenin	Flavanone	Wistar Rats	20, 50, and 100 mg/kg (*oral administration*); 56 days	Naringenin restored hyperglycemia, down-regulated superoxide dismutase activity, and reversed chemical and thermal hyperalgesia in the diabetic rats, showing its preventive and therapeutic effectiveness in diabetic neuropathy treatment.	[101]
Diosmin	Flavone	Sprague-Dawley Rats	50 and 100 mg/kg (*oral administration*); 28 days	Diosmin significantly restored the blood glucose levels, antioxidant parameters, and lipid profiles in the diabetic rats. It also improved their thermal hyperalgesia, cold allodynia, and walking function.	[34]
Sudachitin	Flavone	C57BL/6 J and db/db Mice	5 mg/kg (*oral administration*); 84 days	Sudachitin significantly improved dyslipidemia, reduced triglyceride and free fatty acid contents, enhanced glucose tolerance, and reduced insulin resistance in the diabetic mice. β-oxidation of fatty acids was also markedly enhanced via increased mitochondrial biogenesis.	[54]
Tangeretin	Flavone	Wistar Rats	25, 50, and 100 mg/kg (*intragastric)*; 30 days	Tangeretin normalized the levels and activities of plasma glucose, insulin, glycosylated hemoglobin, and key enzymes of carbohydrate metabolism in the livers of diabetic rats.	[71]
Hesperidin	Flavanone	Sprague-Dawley Rats	25, 50, and 100 mg/kg (*oral administration*); 28 days	Hesperidin decreased the levels of STZ-induced hyperglycemia and pro-inflammatory cytokines and increased the nociceptive threshold, motor nerve conduction velocity, sensory nerve conduction velocity, insulin levels, and Na-K-adenosine triphosphate (ATP)ase activity in the diabetic rats.	[85]
Naringenin	Flavanone	C57BL/6J Mice	100 mg/kg (*oral administration*); 14 days	Naringenin suppressed macrophage infiltration into the adipose tissues of the high-fat diet (HFD)-fed obese mice. It also down-regulated monocyte chemoattractant protein-1 (MCP-1) in the adipose tissues via inhibiting the c-Jun NH2-terminal kinase (JNK) pathway.	[55]
Neohesperidin	Flavanone	KK-Ay and C57BL/6 Mices	50 mg/kg (*oral administration*); 42 days	Neohesperidin attenuated fasting blood glucose and insulin resistance. The levels of total cholesterol, triglycerides, and leptins were significantly decreased, while the phosphorylation of AMP-activated protein kinase (AMPK) and its target genes was increased in the drug-treated mice. It also significantly decreased the size of epididymal adipocytes in the diabetic mice.	[102]
Hesperidin and naringin	Flavanones	Wistar Rats	50 mg/kg (*oral administration*); 30 days	Hesperidin and naringin significantly reduced the glucose level, restored the altered parameters of glucose metabolism, and enhanced adipose tissue glucose transporter type 4 (GLUT4) mRNA and protein expression in the diabetic rats.	[56]
Tangeretin	Flavone	Wistar Rats	100 mg/kg (*intragastric*); 30 days	Tangeretin significantly reduced plasma and cardiac lipid profiles by regulating key lipid metabolic enzymes in the livers of diabetic rats. It also markedly restored the GLUT4 expression, antioxidant enzyme activities, and levels of inflammatory cytokines in the heart tissues of the the tangeretin-treated diabetic rats.	[72]
Nobiletin	Flavone	Wistar rats	10 and 25 mg/kg (*oral administration*); 28 days	Nobiletin substantially ameliorated hemodynamic parameters, oxidative stress, collagen levels, matrix metalloproteinase (MMP)-2 levels, and MMP-9 levels in the diabetic rats. It also markedly attenuated deterioration in the morphology of cardiomyocytes.	[44]
Nobiletin	Flavone	C57BL/6 Mice	50 mg/kg (*oral administration*); 77 days	Nobiletin significantly decreased the expression of nicotinamide adenine dinucleotide (NADH) oxidase isoforms p67phox, p22phox, and p91phox, and attenuated oxidative stress in diabetic mice. It also ameliorated the development of cardiac dysfunction and interstitial fibrosis by down-regulating the c-Jun N-terminal kinase (JNK), P38, and nuclear factor kappa B NF-κB signaling pathways.	[45]
Diosmin	Flavone	Sprague-Dawley Rats	160 mg/kg (*intraperitoneal*); 7 days	Diosmin reduced hyperglycemia by enhancing the secretion of β-endorphin from the adrenal glands via imidazoline 1–2 receptor (I-2R) activation, which triggered the opioid receptors to attenuate gluconeogenesis metabolism in the livers of diabetic rats. It decreased the hepatic glycogen content and plasma lipid profiles in STZ-induced diabetic rats. However, it did not adversely affect the body weight, food intake, and plasma insulin level in the diabetic rats.	[35]
Hesperidin and quercetin	Flavanone and flavone	Wistar Rats	100 mg/kg (*oral administration*); 15 days	Hesperidin and quercetin exerted positive effects on insulin metabolism. They lowered the levels of triglycerides, MDA, TNFα, and IL-6, and restored the level of glutathione (GSH) in experimental diabetic rats induced by STZ.	[87]
Hesperidin	Flavanone	Sprague-Dawley Rats	25, 50, and 100 mg/kg (*oral administration*); 21 days	Hesperidin ameliorated the increased levels of blood glucose, serum insulin, food intake, and water intake in STZ- induced diabetes. It also had a protective effect on the wound architecture by accelerating angiogenesis and vasculogenesis via the up-regulation of vascular endothelial growth factor c (VEGF-c), Angiopoietin (Ang)-1/Tie-2, transforming growth factor (TGF-β), and small mothers against decapentaplegic (Smad)-2/3 mRNA expression to enhance wound healing in the chronic diabetic foot ulcer condition in the diabetic rats.	[86]
Xanthohumol and 8-prenylnaringenin	Prenylflavonoid	C57Bl/6 Mice	0.1% of flavonoids dissolved in ethanol; 140 days	Xanthohumol and 8-prenylnaringenin have a potent therapeutic effect on diabetic mice, as evidenced by the decreased levels of diabetes-linked biochemical parameters in the liver and kidney. They also decreased the overexpression of galectin-3 (Gal3), which was correlated with oxidative stress in diabetic mice.	[27]
Naringin	Flavanone	Sprague-Dawley Rats	100 mg/kg (*oral administration*); 28 days	Naringin reduced blood glucose, total cholesterol, triglycerides, and low-density lipoproteins in fructose-fed rats. Naringin restored acetylcholine-mediated vasorelaxation, suggesting its potential influence on fructose-induced metabolic alterations and endothelial dysfunction. Naringin improved serum nitrate/nitrite (NOx), endothelial nitric oxide synthase (eNOS), and phosphorylated eNOS (p-eNOS) protein expression, and preserved endothelium-dependent relaxation in the aortae of the fructose-fed rats.	[103]
Hesperetin	Flavanone	Wistar Rats	40 mg/kg (intragastric); 45 days	Hesperetin reduced the blood glucose level and enhanced the plasma insulin and the hepatic glycogen levels in the STZ-induced diabetic rats. It also restored the altered hepatic glucose metabolic enzymes, lipid profiles, and serum biomarkers, and protected from STZ-mediated structural alterations and functional changes in the liver, kidneys, and pancreatic β-cells of diabetic animals.	[97]
Hesperetin	Flavanone	Wistar Rats	50 mg/kg (*oral administration*); 46 days	Hesperetin significantly reduced the serum glucose level and improved the serum testosterone level in the STZ-induced diabetic rats. Additionally, it augmented the testicular antioxidant enzymes and attenuated the testicular inflammatory markers, such as TNFα and IL-17, besides preventing the seminiferous tubules’ damage in diabetic rats.	[98]
Naringenin	Flavanone	C57BL/6J Mice	100 mg/kg (*oral administration*); 14 days	Naringenin inhibited neutrophil infiltration into the adipose tissues of the high-fat diet (HFD)-fed mice by reducing the expression of several chemokines, including monocyte chemoattractant protein (MCP)-1 and MCP-3, in the adipose tissues.	[64]
Nobiletin	Flavone	Ldlr-/- and Ampkβ1-/- mice from a C57BL/6J background	0.3% of nobiletin mixed in HFD; 84–126 days	Nobiletin attenuated obesity, hepatic steatosis, dyslipidemia, and insulin resistance, and improved energy utilization in HFD-fed mice. It conferred metabolic protection independently of AMPK activation in the liver and adipose tissues.	[46]

### 2.11. Naringenin

Naringenin (5,7-dihydroxy-2- (4-hydroxyphenyl) chroman-4-one) is a citrus flavanone mainly found in grapefruits, bergamots, and oranges. Numerous pharmacological activities of naringenin have already been reported in the scientific literature. It is widely used as a dietary supplement in different treatments, often in combination with other herbal preparations. Naringenin (aglycone) and naringin are flavanones that display strong anti-inflammatory and antioxidant activities [104].

Since cardiovascular diseases (CVDs) remain the leading cause of morbidity and mortality in diabetic patients, Fallahi, Roghani, and Moghadami [100] focused their study on investigating the cardiovascular potential of this natural flavonoid in diabetes. More specifically, the authors investigated its aortic reactivity, since increased serum glucose and ROS cause vascular endothelial dysfunction. Naringenin prevented weight loss and lowered the increased plasma glucose concentration in diabetic animals, suggesting its cardioprotective effects. This bioflavonoid exhibited beneficial effects on the cardiovascular system by reducing the maximum contractile response of endothelium-intact rings and improving endothelium-dependent relaxation in response to acetylcholine (ACh). These effects seem to be dependent on modulating the NO pathway, since the pretreatment of endothelium-intact rings with the NOS inhibitor N (G)-nitro-l-arginine methyl ester (L-NAME) significantly attenuated the observed responses in diabetic rats.

Hasanein and Fazeli [101] also investigated the antidiabetic effects of naringenin but with a special focus on its effectiveness in diabetes-induced hyperalgesia and allodynia. Naringenin attenuated chemical and thermal hyperalgesia, as well as allodynia. Since oxidative stress and inflammation are also found in diabetic neuropathy, the study showed that naringenin administration increased the activity of SOD, an endogenous enzyme closely intertwined with oxidative stress during the diabetic condition. 

Furthermore, using an HFD-induced obesity animal model (C57BL/6J Mice), Yoshida et al. [55] evaluated the anti-inflammatory effects of naringenin and its mechanism of action. Naringenin did not affect HFD-induced changes in serum biochemical parameters, such as glucose, TC, and TG levels. However, it was able to reduce the mRNA expression of the Mac-2 gene, an important macrophage marker. Reinforcing its anti-inflammatory effect, the administration of naringenin reduced monocyte chemoattractant protein 1 (MCP-1) expression in adipose tissue from HFD-fed mice, and in adipocyte and macrophage co-cultures, which is one of the key chemokines, one of its main roles being to suppress the migration and infiltration of monocytes/macrophages into adipose tissue. In addition, naringenin inhibited HFD-induced JNK phosphorylation but did not interfere in the expression of IκB-α, a member of a family of cellular proteins that function to inhibit the NF-κB transcription factor. In summary, these results suggest that naringenin suppresses macrophage infiltration and can modulate the chemoattraction of inflammatory cells via the regulation of MCP-1 expression in adipocytes via a JNK-dependent pathway in obesity-related metabolic disorders.

Tsuhako, Yoshida, Sugita, and Kurokawa [64] conducted an in vivo experiment with a HFD-induced obese and insulin-resistant animal model, as well as with in vitro assays, using 3T3-L1 (adipocytes) and RAW264 (macrophages) cells to confirm their hypothesis that naringenin has effects on inflammatory cell infiltration into adipose tissue, in addition to being able to modulate vital chemokines and cytokines. The recruitment of immune cells was observed in obese adipose tissue, which contributes to the initiation and progression of obesity-linked diseases, such as insulin resistance and type 2 diabetes mellitus. They showed that naringenin suppressed the neutrophil infiltration into adipose tissue in obese mice. Naringenin also produced an anti-inflammatory response in the adipose tissues in mice by reducing the levels of the chemokines and/or cytokines MCP-1, macrophage inflammatory protein (MIP)-1α, MIP-2, and MCP-3 and causing a noticeable reduction in the pro-inflammatory cytokine IL-6, although TNF-α was not affected. In the in vitro analyses, naringenin significantly reduced MCP-3 expression at the transcriptional and secretion levels in 3T3-L1 adipocytes, as well as in a co-culture of 3T3-L1 adipocytes and RAW264 macrophages. Thus, the authors suggest that naringenin suppresses neutrophil infiltration into adipose tissue via the regulation of vital inflammatory mediators connected to immune-cell functions.

Similar to other citrus flavonoids, naringenin has also been observed to be a potent NF-κB pathway regulator that directly leads to the obstruction of ROS accumulation due to its ability to act as a scavenger of free radicals and up-regulate the activity of both prooxidant and antioxidant enzymes, which is the most remarkable dual property of this flavonoid [55]. In addition, recent findings also point out that naringenin can down-regulate vital chemokines, which have a significant role in the recruitment and infiltration of inflammatory cells into adipose tissue, and stop the advancement of metabolic disorders, such as insulin resistance and type 2 diabetes mellitus.

### 2.12. Naringin

Naringin is a flavanone-7-O-glycoside located between the flavanone naringenin and the disaccharide neohesperidose. It occurs naturally in citrus fruits, predominantly in grapefruits. Similarly to naringenin, naringin is widely sold as a food supplement because of its cardioprotective, neuroprotective, and immunomodulatory properties [105]. When ingested by humans, naringin is metabolized by naringinase in the liver, so the main product of this metabolism is naringenin, which seems to be responsible, at least in part, for the biological effects attributed to this biomolecule. Naringin showed beneficial effects in acute and chronic models, such as those of diabetic neuropathy [106], pleurisy [107], asthma [108,109], cancer [110], behavioral deficits [111], Alzheimer’s disease [112], and chronic fatigue [113], and in experimental models for inflammation and oxidative stress induced by cisplatin [114].

Mahmoud et al. [83] conducted two studies, in 2012 and 2015, to evaluate the antidiabetic potential of naringin. According to the authors, naringin attenuated hyperglycemia-mediated oxidative stress parameters (MDA and NO) and pro-inflammatory cytokine (TNF-α and IL-6) secretion and production in HFD/STZ-induced type 2 diabetic rats. The authors confirmed that the oral administration of naringin normalized the activities of important enzymes in hepatic glycolytic metabolism, such as G6Pase, glycogen phosphorylase, and fructose-1,6-bisphosphatase. In addition, naringin increased the release of insulin from isolated islets in the presence of IL-1β and decreased intestinal glucose absorption. Its antioxidant effects were also verified due to the reduction of NO in isolated pancreatic islets. The mechanism of the action responsible for the effects of naringin may be related to the increased expression of GLUT4 in adipose tissue, which aids in the uptake of free circulating glucose from the blood to peripheral tissues. 

Malakul, Pengnet, Kumchoom, and Tunsophon [103] investigated the effect of naringin on fructose-induced endothelial dysfunction in rats and its fundamental mechanisms. Rats that had consumed fructose in drinking water showed significantly increased levels of blood glucose, TC, TG, and LDL C. Consequently, naringin treatment significantly brought these parameters back to near normal. Fructose impaired endothelial function, but vascular smooth muscle function was unaffected by fructose treatment. Interestingly, naringin restored endothelial function in the aortic rings, confirming a vasoprotective effect. In addition, naringin improved serum nitrate/nitrite (NOx), eNOS, and phosphorylated eNOS (p-eNOS) protein expression. Therefore, the authors concluded that the vascular potential of naringin was moderately attributed to improving NO bioavailability, increasing eNOS activity, and obstructing the accumulation of peroxynitrite in the aortae.

In summary, the results of these studies demonstrate the related curative potential of naringin in attenuating oxidative damage and inflammatory cascades. Moreover, it exhibits a unique preserving effect on endothelial dysfunction, an important factor in the development of diabetic complications, especially atherosclerosis and cardiovascular diseases.

### 2.13. Neohesperidin

Neohesperidin (hesperetin-7-neohesperidoside) is a flavanone glycoside, a weak-polar molecule with a bitter taste, found in various citrus fruits [115]. Neohesperidin, a dihydrochalcone, is a substance mainly obtained from bitter oranges and has unique properties such as masking undesirable flavors and enhancing fruity and citrus flavors, which gives this molecule great value for the food industry and nutraceuticals firms [116]. This flavanone has a wide range of biological activities, including neuroprotective activity [117] and anti-proliferative effects [118]. Recently, neohesperidin was found to inhibit common allergic responses in vivo and in vitro [119], exhibit protective effects in progressive pulmonary fibrosis [120], and show anti-osteoclastic properties, presenting it as a potential anti-catabolic biomolecule for the treatment of osteoporosis [121].

The antidiabetic potential of neohesperidin was investigated by Jia et al. [102], who evaluated the effect of this active compound derived from *Citrus aurantium* Linn. in diabetic KK-Ay mice induced via a formulated diet (6.0% fat, 18% proteins, and 8.0% water). Neohesperidin had no significant effect on the body weight and food intake in the experimental diabetic mice; nevertheless, it increased glucose tolerance and insulin sensitivity and reduced the blood glucose levels affected by diabetic illness. Neohesperidin treatment also significantly reduced total cholesterol and TG, in addition to decreasing ALT, but it did not modulate AST levels, showing its key hypoglycemic and hypolipidemic properties.

Histological studies showed that neohesperidin-treated diabetic mice had a marked reduction in lipid accumulation in the liver and decreased adipocyte size compared with water-treated KK-Ay diabetic mice. Neohesperidin was shown to have hypolipidemic effects via exerting a profound influence on markers, such as the mRNA levels of PPAR-α, PPAR-γ, and their target genes, including stearoyl-CoA desaturase (SCD)-1, carnitine palmitoyltransferase (CPT)-1, adaptor complex (AP)-2, UCP-2, fatty acid synthase (FAS), and acyl-CoA oxidase (ACOX), in liver tissue. The expression of SCD-1 and FAS in diabetic mice was significantly down-regulated by neohesperidin treatment, whereas the expression of ACOX was significantly up-regulated. Finally, neohesperidin treatment resulted in the increased phosphorylation of AMPK. These data demonstrate that neohesperidin may have pronounced potential for the prevention of obesity-linked diabetes mellitus [102].

### 2.14. Xanthohumol

Xanthohumol or 3′-[3,3-dimethylallyl]-2′,4′,4-trihydroxy-6′-methoxychalcone, found in citrus plants in the family Rutaceae, is a bioactive antioxidant molecule linked to a wide range of bioactivities, including anticarcinogenic, anti-inflammatory, and antioxidant properties [122,123]. A study included in our survey demonstrated that xanthohumol reduced the expression of Gal3, a protein responsible for multiple complications and diabetic progression in HFD-fed type 2 diabetic C57Bl/6 mice. In addition to reducing Gal3 expression, xanthohumol has also been shown to reduce oxidative stress biomarkers associated with diabetes such as 3-nitrotyrosine and AGEs in the liver and kidneys, validating its remedial effect against this chronic metabolic disease [27].

### 2.15. Quercetin

This citrus flavonoid is probably one of the most studied flavonol compounds, which may be due to its ubiquitous presence in different citrus plants. It also has many therapeutic properties that include anti-inflammatory, antinociceptive, and anticancer effects. Oranges, mandarins, limes, lemons, sour oranges, and grapefruits are common sources of quercetin [124]. Quercetin is, therefore, considered the most important citrus flavonoids because of its ability to modulate the essential inflammatory mediators that accompany metabolic diseases. This flavonol is one of the most popular citrus flavonoids in the global fruit market, and it is commonly used as a constituent in nutraceuticals and food supplements. A number of products containing this flavonoid have been patented because of its outstanding therapeutic applicability as a disease-fighting antioxidant molecule that can improve the health and well-being of individuals [125].

Dhanya, Arya, Nisha, and Jayamurthy [57] investigated the molecular mechanism of quercetin by screening it in skeletal muscle (L6 myotubes) cells and showed that it improved glucose uptake via regulation of the AMPK pathway. The authors demonstrated that the AMPK pathway has a significant role in 2-NBDG (2-deoxy-2-[(7-nitro-2,1,3-benzoxadiazol-4-yl)amino]-D-glucose) uptake and that this was induced by quercetin. The adenosine monophosphate/adenosine triphosphate ratio (AMP/ATP) is an essential factor for cellular AMPK activation, and quercetin pretreatment caused an increase in both the AMP-to-ATP ratio and the adenosine diphosphate (ADP)-to-ATP ratio, an effect correlated with its activity on mitochondrial membrane depolarization. In addition, quercetin pretreatment in L6 myotubes induced a significant up-regulation of the mRNA levels of both AMPK and its downstream target p38 MAPK. Interestingly, calcium-calmodulin mediated protein kinase (CaMKK), AMPK, and MAPK, the key signaling molecules involved in the AMPK signaling pathway, were up-regulated by quercetin treatment in vitro, reinforcing the evidence of its participation in this vital signaling pathway related to the management of insulin signaling. In addition, quercetin was found to increase GLUT4 expression and translocation in a skeletal muscle cell line. Therefore, quercetin possesses antidiabetic potential via activating multiple therapeutic targets to rectify insulin resistance through bypassing different metabolic pathways.

However, Dokumacioglu et al. [87] have reported some controversial results regarding the effect of quercetin on diabetes. Although quercetin treatment decreased various diabetes-related biochemical parameters, such as TC, TG, LDL C, VLDL C, and MDA, it did not alter the HDL-C level and the GSH concentration. Histological analysis showed that treatment with quercetin led to an increase in the regeneration of β-cells in the pancreatic islets. However, it was also reported that quercetin administration in diabetic animals regulated the levels of pro-inflammatory cytokines, such as TNF-α and IL-6. In addition, quercetin also blocked the weight loss in diabetic rats, which could be the result of quercetin regularizing TNF-α and IL-6 secretion in adipose cells and the consequent decrease in fat tissue.

### 2.16. Rutin

Rutin (quercetin 3-rutinoside, C_27_H_30_O_16_) is a glycosidic flavonoid commonly present in dietary sources. The main citrus fruit sources of rutin are oranges, grapefruits, lemons, and limes. Treatment with rutin was found to exert a great modulatory impact on the secretion of IL-1β, IL-2, IL-4, and IL-6. These are vital immunomodulatory cytokines secreted by the immune cells, and abnormal levels of secretion and their action during inflammation can cause cytokine-mediated organ dysfunction and tissue damage during long-term diseases such as diabetes mellitus, cancer, and rheumatoid arthritis [126]. Its immunomodulatory and anti-inflammatory effects on chronic metabolic disorders characterized by hyperglycemia suggest that rutin may be an excellent biomolecule to use in the treatment of these disorders and their associated complaints.

Fernandes et al. [99] investigated the benefit of rutin treatment for various biochemical alterations in experimental diabetic animals. Rutin reduced blood glucose and improved the lipid profile. In addition, it prevented changes in the activities of ALT, AST, and LDH in the serum, liver, and heart, indicating the protective effect of the molecule against hepatic and cardiac toxicity in diabetic rats. Rutin was also able to decrease hepatic and cardiac levels of TG and elevate the glycogen concentration. It showed hypoglycemic and hypolipidemic effects in diabetic rats and, more importantly, prevented liver and heart damage caused by the uncontrolled accumulation of diabetes-mediated ROS.

Besides the single flavonoid compound mentioned above, the citrus extract, rich in different flavonoids, also shows an antidiabetic effect. A study by Kim et al. [53] investigated the effects of *Citrus aurantium* Linn., which contains major flavonoids including naringin, hesperidin, poncirin, isosiennsetin, sineesytin, nobiletin, and tangeretin, on the inhibition of adipogenesis and adipocyte differentiation in 3T3-L1 cells. The mixed actions of the flavonoids from *C. aurantium* showed anti-adipogenic properties and inhibited the differentiation of 3T3-L1 preadipocytes into adipocytes, in addition to also reducing the amount of lipid droplets, and preventing lipid and triglyceride accumulation. Flavonoid-rich *C. aurantium* was able to modulate the insulin signaling cascade, via the inhibition of Akt activation and GSK3β phosphorylation. Finally, it down-regulated the expression of C/EBPβ and subsequently inhibited the activation of PPARγ and C/EBPα, which are related to lipid accumulation and lipid metabolism. Thus, these results highlight the actions of these vital flavonoids present in *C. aurantium* in improving hyperglycemia and dyslipidemia, while inhibiting the progression of diabetes. 

## 3. Composition of Antidiabetic Citrus Flavonoids in Common Citrus Fruit Sources

Few review reports and original articles concern the composition of some major flavonoids in the genus *Citrus.* In our survey, the majority of the antidiabetic studies reported used citrus flavanones (hesperidin, didymin, naringin, neohesperidin, and poncirin), flavones (rhoifolin, diosmin, and rutin), and polymethoxyflavones (nobiletin, sinesetin, and tangeretin). The flavonoid composition of long-life orange juice comprises hesperidin (76.9 mg/L) and didymin (9.9 mg/L) [127]. The composition of tangerine or mandarin hand-squeezed juice comprised didymin (4.44–9.50 mg/L), hesperidin (123.3–206.7 mg/L), tangeritin (5.99–31.8 mg/L), nobiletin (5.49–28.2 mg/L), and sinensetin (0.30–2.00 mg/L), whereas the peeled fruit of tangerines or mandarins contained higher contents of hesperidin (841–1898 mg/kg) and didymin (45–112 mg/kg) [128]. The flavonoid contents in fresh weight (FW) peels and peel extracts of citrus fruits, such as oranges, clementines, and mandarins, were poncirin (2.49–18.85 mg/g FW), didymin (3.22–13.94 mg/g FW), neohesperidin (3.20–11.67 mg/g FW), hesperidin (83.4–234.1 mg/g FW), naringin (only in mandarin) (19.49 mg/g FW), rhoifolin (4.54–10.39 mg/g FW), diosmin (4.01–18.06 mg/g FW), and rutin (8.16–42.13 mg/g FW) [129].

In addition, Gattuso et al. [130] have reviewed the flavonoid contents in various citrus juices extensively. For example, sweet orange juice contains hesperidin (28.6 mg/100 mL), didymin (1.89 mg/100 mL), poncirin (1.04 mg/100 mL), rhoifolin (0.05 mg/100 mL), diosmin (0.09 mg/100 mL), nobiletin (0.33 mg/100 mL), sinesetin (0.37 mg/100 mL), and tangeretin (0.04 mg/100 mL); clementine juice possess hesperidin (39.9 mg/100 mL), naringin (0.08 mg/100 mL), and diosmin (1.25 mg/100 mL); lemon juice has hesperidin (20.5 mg/100 mL) and diosmin (3.12 mg/100 mL); grapefruit juice contains didymin (0.30 mg/100 mL), hesperidin (0.93 mg/100 mL), naringin (23.0 mg/100 mL), neohesperidin (1.21 mg/100 mL), poncirin (1.26 mg/100 mL), rutin (3.26 mg/100 mL), rhoifolin (0.28 mg/100 mL), nobiletin (0.15 mg/100 mL), tangeretin (0.12 mg/100 mL), hesperetin (0.74 mg/100 mL), naringenin (2.70 mg/100 mL), and quercetin (0.19 mg/100 mL); the juice of a hybrid between lemon and sweet oranges contains naringin (2.23 mg/100 mL), neohesperidin (1.60 mg/100 mL), poncirin (6.41 mg/100 mL), rhoifolin (0.37 mg/100 mL), and diosmin (0.39 mg/100 mL); mandarin orange juice has didymin (1.44 mg/100 mL), hesperidin (24.3 mg/100 mL), nobiletin (0.23 mg/100 mL), sinensetin (1.05 mg/100 mL), and tangeretin (0.26 mg/100 mL); hybrid mandarin orange juice contains hesperidin (0.15 mg/100 mL); and bitter orange juice has naringin (1.97 mg/100 mL), neohesperidin (0.87 mg/100 mL), poncirin (0.73 mg/100 mL), diosmin (0.15 mg/100 mL), nobiletin (0.2 mg/100 mL), and tangeretin (0.08 mg/100 mL).

However, few studies have reported the relationships between the antidiabetic effects and the structure of citrus flavonoids, which should be investigated in the future to elucidate their structure–function relationships.

## 4. Conclusions

**Figure 3:**
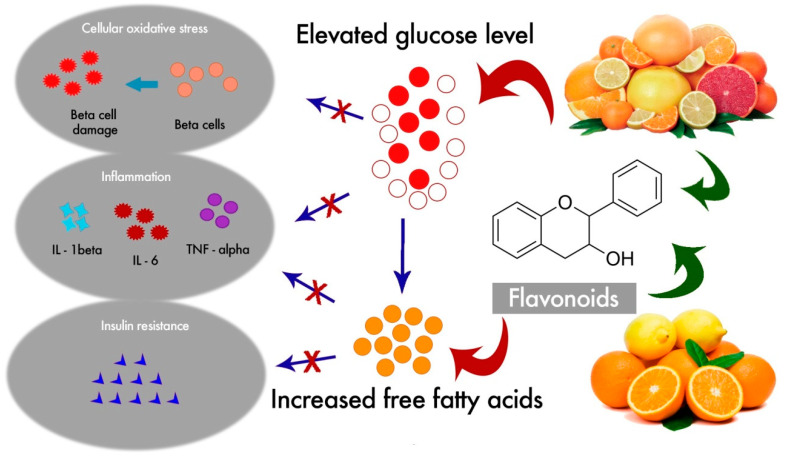
Proposed antidiabetic mechanisms of action of citrus flavonoids. The pictorial representation summarizes the current knowledge that citrus flavonoids could improve the pathogenesis of diabetes and its complications via attenuating cellular oxidative stress, inflammatory markers (interleukin (IL) -1beta, IL-6, tumor necrosis factor (TNF)-alpha), and insulin resistance.

**Figure 4:**
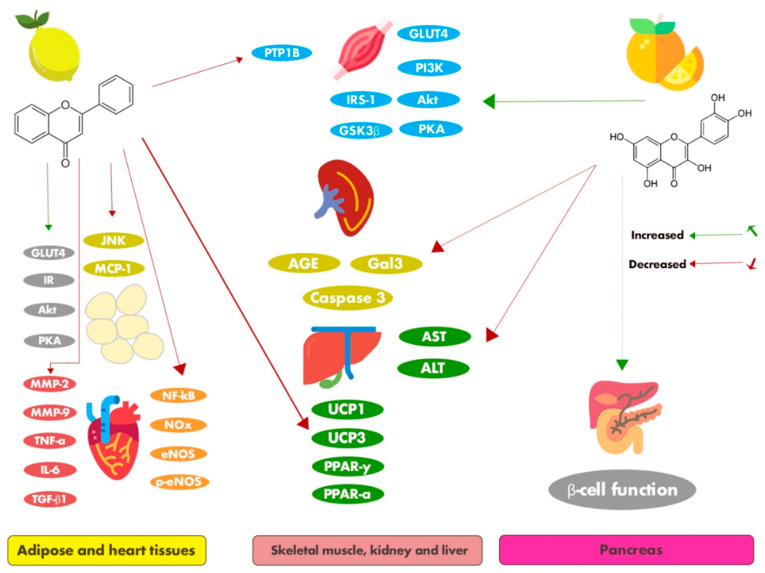
Citrus flavonoids target several molecular markers that are involved in the regulation of blood glucose levels. Citrus flavonoids can increase adipose tissue glucose transporter 4 (GLUT4), insulin receptors (IR), protein kinase B (PKB) or Akt, and protein kinase A (PKA); decrease skeletal muscle protein tyrosine phosphatase 1B (PTP1B); and up-regulate GLUT4, phosphoinositide 3-kinases (PI3K), insulin receptor substrate (IRS)-1, Akt, PKA, and glycogen synthase kinase 3β (GSK3β) expression in the skeletal muscle tissue. They also improve β-cell function. On the other hand, citrus flavonoid molecules can decrease c-Jun N-terminal kinase (JNK) and monocyte chemoattractant protein (MCP)-1 in the adipose tissue, and down-regulate nuclear factor kappa B (NF-κB), nitrate/nitrite (NOx), endothelial nitric oxide synthase (eNOS), matrix metalloproteinases (MMPs), and inflammatory mediators in the heart tissue. They also reduce the glycation end products (AGEs), Galectin-3 (Gal3), and caspase 3 expression in the kidney and decrease the uncoupling protein (UCP), proliferator-activated receptor (PPAR), aspartate aminotransferase (AST), and alanine aminotransferase (ALT) levels in the liver tissue.

In conclusion, using bioactive molecules from plant dietary sources is a fascinating therapeutic process but requires detailed knowledge of their effects drawn from different types of experimental models when used in the treatment of various syndromes. In this review study, the 19 flavonoids of the Citrus genus surveyed present diverse effects and related molecular mechanisms for the treatment and management of diabetic mellitus and related complications. The related antidiabetic mechanisms of citrus flavonoids are illustrated in Figure 3 and Figure 4. These citrus flavonoids attenuated tissue damage arising from prolonged exposure to elevated glucose levels, mainly by increasing endogenous antioxidants, such as SOD, CAT, and GPx, and reducing the concentration of ROS. Regarding the key molecular mechanisms, citrus flavonoids modulate vital metabolic signaling markers via increasing the expression of IRS-1, PI3K, GSK3β, Akt, and PPARγ, and decreasing the expression of PTP1B. The citrus flavonoids are also involved in activating and increasing the expression of the imidazoline I-2R, opioid secretion, GLUT4, and IR, and they also modulate the expression of eNOS, MCP-1 and 3, NF-κB, the cytokines TNFα and INFγ, IL1β, IL-2, and IL-6. All of these processes result in the attenuation of inflammatory mediators linked to the pathogenesis and progression of diabetic vascular complications by increasing glucose uptake in peripheral tissues. This also has a preventive effect against high glucose-induced cell proliferation.

In the future, more detailed research is still required into these compounds, along with the development of various drug delivery vehicles that facilitate their controlled release and increase their absorption, bioavailability, and potency. Conducting human clinical trials is the only fool-proof method for determining the efficacy of citrus flavonoids in humans. Therefore, it is important to undertake human clinical trials based on the current knowledge about these compounds using modern molecular technological tools to identify the mechanisms of action in different pathways and molecular gene expression studies using type 2 diabetes-related genes. 

Moreover, easier and cheaper methods to isolate pure compounds should be further explored so that comprehensive studies can be conducted on every potential antidiabetic citrus flavonoid. Emphasis must be given to the citrus flavonoids as safer, novel antidiabetic agents, rather than being overly dependent on synthetic antidiabetic drugs. Accurate scientific information on citrus flavonoids should be collected and distributed as widely as possible through publications and seminars. 

Overall, the Citrus species are valuable natural sources of flavonoids and a promising source for future treatments aimed at the prevention and management of diabetes and related complications. To the best of our knowledge, this is the first review that summarizes the effects of major citrus flavonoids on the vital physiological pathways and biochemical parameters related to diabetes. This can contribute to the understanding of their biological profiles in current therapies and assist in the development of future therapies for the treatment of diabetes.

## References

[1] Zech-Matterne V., Fiorentino G. (2017). AGRUMED: Archaeology and History of Citrus Fruit in the Mediterranean: Acclimatization, Diversifications, Uses.

[2] Luro F., Gatto J., Costantino G., Pailly O. (2011). Analysis of genetic diversity in *Citrus*. Plant Genet. Resour..

[3] Goulet B.E., Roda F., Hopkins R. (2017). Hybridization in plants: Old ideas, new techniques. Plant Physiol..

[4] Ollitrault P., Navarro L., Badenes M., Byrne D. (2012). Citrus. Fruit Breeding.

[5] Gill H., Garg H. (2017). Citrus Pathology.

[6] Man M.Q., Yang B., Elias P.M. (2019). Benefits of hesperidin for cutaneous functions. Evid. Based Complement. Alternat. Med..

[7] Tripoli E., Guardia M.L., Giammanco S., Majo D.D., Giammanco M. (2007). *Citrus* flavonoids: Molecular structure, biological activity and nutritional properties: A review. Food Chem..

[8] Mahmoud A.M., Hernández Bautista R.J., Sandhu M.A., Hussein O.E. (2019). Beneficial effects of citrus flavonoids on cardiovascular and metabolic health. Oxid. Med. Cell. Longev..

[9] Diabetes Atlas 9th edition 2019. https://www.diabetesatlas.org/en/.

[10] Reach G., Pechtner V., Gentilella R., Corcos A., Ceriello A. (2017). Clinical inertia and its impact on treatment intensification in people with type 2 diabetes mellitus. Diabetes Metab..

[11] Cho N.H., Shaw J.E., Karuranga S., Huang Y., da Rocha Fernandes J.D., Ohlrogge A.W., Malanda B. (2018). IDF Diabetes Atlas: Global estimates of diabetes prevalence for 2017 and projections for 2045. Diabetes Res. Clin. Pract..

[12] Gray S.P., Jandeleit-Dahm K. (2014). The pathobiology of diabetic vascular complications—Cardiovascular and kidney disease. J. Mol. Med..

[13] Wong E., Backholer K., Gearon E., Harding J., Freak-Poli R., Stevenson C., Peeters A. (2013). Diabetes and risk of physical disability in adults: A systematic review and meta-analysis. Lancet Diabetes Endocrinol..

[14] Heinonen S.E., Genové G., Bengtsson E., Hübschle T., Åkesson L., Hiss K., Benardeau A., Ylä-Herttuala S., Jönsson-Rylander A.-C., Gomez M.F. (2015). Animal models of diabetic macrovascular complications: Key players in the development of new therapeutic approaches. J. Diabetes Res..

[15] Zhang P., Li T., Wu X., Nice E.C., Huang C., Zhang Y. (2020). Oxidative stress and diabetes: Antioxidative strategies. Front. Med..

[16] Yaribeygi H., Sathyapalan T., Atkin S.L., Sahebkar A. (2020). Molecular mechanisms linking oxidative stress and diabetes mellitus. Oxid. Med. Cell. Longev..

[17] Li C., Schluesener H. (2017). Health-promoting effects of the citrus flavanone hesperidin. Crit. Rev. Food Sci. Nutr..

[18] Millar C.L., Duclos Q., Blesso C.N. (2017). Effects of dietary flavonoids on reverse cholesterol transport, HDL metabolism, and HDL function. Adv. Nutr..

[19] Rees A., Dodd G.F., Spencer J.P.E. (2018). The effects of flavonoids on cardiovascular health: A review of human intervention trials and implications for cerebrovascular function. Nutrients.

[20] Zaidun N.H., Thent Z.C., Latiff A.A. (2018). Combating oxidative stress disorders with citrus flavonoid: Naringenin. Life Sci..

[21] Zhang X., Li X., Fang H., Guo F., Li F., Chen A., Huang S. (2019). Flavonoids as inducers of white adipose tissue browning and thermogenesis: Signalling pathways and molecular triggers. Nutr. Metab..

[22] Kopustinskiene D.M., Jakstas V., Savickas A., Bernatoniene J. (2020). Flavonoids as anticancer agents. Nutrients.

[23] Moher D., Liberati A., Tetzlaff J., Altman D.G. (2009). Preferred reporting items for systematic reviews and meta-analyses: The PRISMA statement. PLoS Med..

[24] Żołnierczyk A.K., Mączka W.K., Grabarczyk M., Wińska K., Woźniak E., Anioł M. (2015). Isoxanthohumol—Biologically active hop flavonoid. Fitoterapia.

[25] Štulíková K., Karabín M., Nešpor J., Dostálek P. (2018). Therapeutic perspectives of 8-prenylnaringenin, a potent phytoestrogen from hops. Molecules.

[26] Negrão R., Costa R., Duarte D., Taveira Gomes T., Mendanha M., Moura L., Vasques L., Azevedo I., Soares R. (2010). Angiogenesis and inflammation signaling are targets of beer polyphenols on vascular cells. J. Cell. Biochem..

[27] Luís C., Costa R., Rodrigues I., Castela Â., Coelho P., Guerreiro S., Gomes J., Reis C., Soares R. (2018). Xanthohumol and 8-prenylnaringenin reduce type 2 diabetes-associated oxidative stress by downregulating galectin-3. Porto Biomed. J..

[28] Petrie J.R., Guzik T.J., Touyz R.M. (2018). Diabetes, hypertension and cardiovascular disease: Clinical insights and vascular mechanisms. Can. J. Cardiol..

[29] Munhoz A.C.M., Frode T.S. (2018). Isolated compounds from natural products with potential antidiabetic activity—A systematic review. Curr. Diabetes Rev..

[30] Rao Y.K., Lee M.J., Chen K., Lee Y.C., Wu W.S., Tzeng Y.M. (2011). Insulin-mimetic action of rhoifolin and cosmosiin isolated from *Citrus grandis* (L.) Osbeck leaves: Enhanced adiponectin secretion and insulin receptor phosphorylation in 3T3-L1 cells. Evid. Based Complement. Alternat. Med..

[31] Shalkami A.S., Hassan M., Bakr A.G. (2018). Anti-inflammatory, antioxidant and anti-apoptotic activity of diosmin in acetic acid-induced ulcerative colitis. Hum. Exp. Toxicol..

[32] Bogucka-Kocka A., Woźniak M., Feldo M., Kocki J., Szewczyk K. (2013). Diosmin—Isolation techniques, determination in plant material and pharmaceutical formulations, and clinical use. Nat. Prod. Commun..

[33] Srinivasan S., Pari L. (2013). Antihyperlipidemic effect of diosmin: A citrus flavonoid on lipid metabolism in experimental diabetic rats. J. Funct. Foods.

[34] Jain D., Bansal M.K., Dalvi R., Upganlawar A., Somani R. (2014). Protective effect of diosmin against diabetic neuropathy in experimental rats. J. Integr. Med..

[35] Hsu C.C., Lin M.H., Cheng J.T., Wu M.C. (2017). Diosmin, a citrus nutrient, activates imidazoline receptors to alleviate blood glucose and lipids in type 1-like diabetic rats. Nutrients.

[36] Huang H., Li L., Shi W., Liu H., Yang J., Yuan X., Wu L. (2016). The multifunctional effects of nobiletin and its metabolites in vivo and in vitro. Evid. Based Complement. Alternat. Med..

[37] Lee Y.-S., Cha B.-Y., Choi S.-S., Choi B.-K., Yonezawa T., Teruya T., Nagai K., Woo J.-T. (2013). Nobiletin improves obesity and insulin resistance in high-fat diet-induced obese mice. J. Nutr. Biochem..

[38] Wang Y., Xie J., Ai Z., Su J. (2019). Nobiletin-loaded micelles reduce ovariectomy-induced bone loss by suppressing osteoclastogenesis. Int. J. Nanomed..

[39] Goh J.X.H., Tan L.T., Goh J.K., Chan K.G., Pusparajah P., Lee L.H., Goh B.H. (2019). Nobiletin and derivatives: Functional compounds from citrus fruit peel for colon cancer chemoprevention. Cancers.

[40] Nohara K., Nemkov T., D‘Alessandro A., Yoo S.H., Chen Z. (2019). Coordinate regulation of cholesterol and bile acid metabolism by the clock modifier nobiletin in metabolically challenged old mice. Int. J. Mol. Sci..

[41] Mulvihill E.E., Assini J.M., Lee J.K., Allister E.M., Sutherland B.G., Koppes J.B., Sawyez C.G., Edwards J.Y., Telford D.E., Charbonneau A. (2011). Nobiletin attenuates VLDL overproduction, dyslipidemia, and atherosclerosis in mice with diet-induced insulin resistance. Diabetes.

[42] Onda K., Horike N., Suzuki T., Hirano T. (2013). Polymethoxyflavonoids tangeretin and nobiletin increase glucose uptake in murine adipocytes. Phytother. Res..

[43] Kanda K., Nishi K., Kadota A., Nishimoto S., Liu M.C., Sugahara T. (2012). Nobiletin suppresses adipocyte differentiation of 3T3-L1 cells by an insulin and IBMX mixture induction. Biochim. Biophys. Acta.

[44] Parkar N.A., Bhatt L.K., Addepalli V. (2016). Efficacy of nobiletin, a citrus flavonoid, in the treatment of the cardiovascular dysfunction of diabetes in rats. Food Funct..

[45] Zhang N., Yang Z., Xiang S.Z., Jin Y.G., Wei W.Y., Bian Z.Y., Deng W., Tang Q.Z. (2016). Nobiletin attenuates cardiac dysfunction, oxidative stress, and inflammatory in streptozotocin: Induced diabetic cardiomyopathy. Mol. Cell. Biochem..

[46] Morrow N.M., Burke A.C., Samsoondar J.P., Seigel K.E., Wang A., Telford D.E., Sutherland B.G., O’Dwyer C., Steinberg G.R., Fullerton M.D. (2020). The citrus flavonoid nobiletin confers protection from metabolic dysregulation in high-fat-fed mice independent of AMPK. J. Lipid Res..

[47] Refaat J., Desoukey S.Y., Ramadan M.A., Kamel M.S. (2015). Rhoifolin: A review of sources and biological activities. Int. J. Pharmacogn..

[48] Liao S., Song F., Feng W., Ding X., Yao J., Song H., Liu Y., Ma S., Wang Z., Lin X. (2019). Rhoifolin ameliorates titanium particle-stimulated osteolysis and attenuates osteoclastogenesis via RANKL-induced NF-κB and MAPK pathways. J. Cell. Physiol..

[49] Phan V.K., Nguyen T.M., Minh C.V., Nguyen H.K., Nguyen H.D., Nguyen P.T., Nguyen X.C., Nguyen H.N., Nguyen X.N., Heyden Y.V. (2010). Two new C-glucosyl benzoic acids and flavonoids from Mallotus nanus and their antioxidant activity. Arch. Pharm. Res..

[50] Cheng L., Ren Y., Lin D., Peng S., Zhong B., Ma Z. (2017). The anti-inflammatory properties of Citrus *wilsonii tanaka* extract in LPS-induced RAW 264.7 and primary mouse bone marrow-derived dendritic cells. Molecules.

[51] Sultana B., Yaqoob S., Zafar Z., Bhatti H.N. (2018). Escalation of liver malfunctioning: A step toward Herbal Awareness. J. Ethnopharmacol..

[52] Koyuncu I. (2018). Evaluation of anticancer, antioxidant activity and phenolic compounds of *Artemisia absinthium* L.. Extract. Cell. Mol. Biol..

[53] Kim G.S., Park H.J., Woo J.H., Kim M.K., Koh P.O., Min W., Ko Y.G., Kim C.H., Won C.K., Cho J.H. (2012). *Citrus aurantium* flavonoids inhibit adipogenesis through the Akt signaling pathway in 3T3-L1 cells. BMC Complement. Altern. Med..

[54] Tsutsumi R., Yoshida T., Nii Y., Okahisa N., Iwata S., Tsukayama M., Hashimoto R., Taniguchi Y., Sakaue H., Hosaka T. (2014). Sudachitin, a polymethoxylated flavone, improves glucose and lipid metabolism by increasing mitochondrial biogenesis in skeletal muscle. Nutr. Metab..

[55] Yoshida H., Watanabe H., Ishida A., Watanabe W., Narumi K., Atsumi T., Sugita C., Kurokawa M. (2014). Naringenin suppresses macrophage infiltration into adipose tissue in an early phase of high-fat diet-induced obesity. Biochem. Biophys. Res. Commun..

[56] Mahmoud A.M., Ahmed O.M., Ashour M.B., Abdel-Moneim A. (2015). In vivo and in vitro antidiabetic effects of citrus flavonoids; a study on the mechanism of action. Int. J. Diabetes Dev. Ctries..

[57] Dhanya R., Arya A.D., Nisha P., Jayamurthy P. (2017). Quercetin, a lead compound against type 2 diabetes ameliorates glucose uptake via AMPK pathway in skeletal muscle cell line. Front. Pharmacol..

[58] Liu W., Liou S.S., Hong T.Y., Liu I.M. (2017). Protective effects of hesperidin (citrus flavonone) on high glucose induced oxidative stress and apoptosis in a cellular model for diabetic retinopathy. Nutrients.

[59] Zareei S., Boojar M.M.A., Amanlou M. (2017). Inhibition of liver alanine aminotransferase and aspartate aminotransferase by hesperidin and its aglycone hesperetin: An in vitro and in silico study. Life Sci..

[60] Chen F., Ma Y., Sun Z., Zhu X. (2018). Tangeretin inhibits high glucose-induced extracellular matrix accumulation in human glomerular mesangial cells. Biomed. Pharmacother..

[61] Shukla K., Sonowal H., Saxena A., Ramana K.V. (2018). Didymin prevents hyperglycemia-induced human umbilical endothelial cells dysfunction and death. Biochem. Pharmacol..

[62] Ali M.Y., Zaib S., Rahman M.M., Jannat S., Iqbal J., Park S.K., Chang M.S. (2019). Didymin, a dietary citrus flavonoid exhibits anti-diabetic complications and promotes glucose uptake through the activation of PI3K/Akt signaling pathway in insulin-resistant HepG2 cells. Chem. Biol. Interact..

[63] Tseng Y.-T., Hsu H.-T., Lee T.-Y., Chang W.-H., Lo Y.-C. (2019). Naringenin, a dietary flavanone, enhances insulin-like growth factor 1 receptor-mediated antioxidant defense and attenuates methylglyoxal-induced neurite damage and apoptotic death. Nutr. Neurosci..

[64] Tsuhako R., Yoshida H., Sugita C., Kurokawa M. (2019). Naringenin suppresses neutrophil infiltration into adipose tissue in high-fat diet-induced obese mice. J. Nat. Med..

[65] Nakagawa H., Takaishi Y., Tanaka N., Tsuchiya K., Shibata H., Higuti T. (2006). Chemical constituents from the peels of *Citrus sudachi*. J. Nat. Prod..

[66] Ohyama Y., Ito J., Kitano V.J., Shimada J., Hakeda Y. (2018). The polymethoxy flavonoid sudachitin suppresses inflammatory bone destruction by directly inhibiting osteoclastogenesis due to reduced ROS production and MAPK activation in osteoclast precursors. PLoS ONE.

[67] Abe S., Hirose S., Nishitani M., Yoshida I., Tsukayama M., Tsuji A., Yuasa K. (2018). Citrus peel polymethoxyflavones, sudachitin and nobiletin, induce distinct cellular responses in human keratinocyte HaCaT cells. Biosci. Biotechnol. Biochem..

[68] Mitani M., Minatogawa Y., Nakamoto A., Nakamoto M., Shuto E., Nii Y., Sakai T. (2019). Sudachitin, polymethoxyflavone from *Citrus sudachi*, enhances antigen-specific cellular and humoral immune responses in BALB/c mice. J. Clin. Biochem. Nutr..

[69] Hosokawa Y., Hosokawa I., Ozaki K., Matsuo T. (2019). Sudachitin inhibits matrix metalloproteinase-1 and -3 production in tumor necrosis factor-α-stimulated human periodontal ligament cells. Inflammation.

[70] Ashrafizadeh M., Ahmadi Z., Mohammadinejad R., Afshar E.G. (2020). Tangeretin: A mechanistic review of its pharmacological and therapeutic effects. J. Basic Clin. Physiol. Pharmacol..

[71] Sundaram R., Shanthi P., Sachdanandam P. (2014). Effect of tangeretin, a polymethoxylated flavone on glucose metabolism in streptozotocin-induced diabetic rats. Phytomedicine.

[72] Sundaram R., Shanthi P., Sachdanandam P. (2015). Tangeretin, a polymethoxylated flavone, modulates lipid homeostasis and decreases oxidative stress by inhibiting NF-κB activation and proinflammatory cytokines in cardiac tissue of streptozotocin-induced diabetic rats. J. Funct. Foods.

[73] Yao Q., Lin M.T., Zhu Y.D., Xu H.L., Zhao Y.Z. (2018). Recent trends in potential therapeutic applications of the dietary flavonoid didymin. Molecules.

[74] Hung J.Y., Hsu Y.L., Ko Y.C., Tsai Y.M., Yang C.J., Huang M.S., Kuo P.L. (2010). Didymin, a dietary flavonoid glycoside from citrus fruits, induces Fas-mediated apoptotic pathway in human non-small-cell lung cancer cells in vitro and in vivo. Lung Cancer.

[75] Singhal J., Nagaprashantha L.D., Vatsyayan R., Awasthi S., Singhal S.S. (2012). Didymin induces apoptosis by inhibiting N-Myc and upregulating RKIP in neuroblastoma. Cancer Prev. Res..

[76] Wei J., Huang Q., Bai F., Lin J., Nie J., Lu S., Lu C., Huang R., Lu Z., Lin X. (2017). Didymin induces apoptosis through mitochondrial dysfunction and up-regulation of RKIP in human hepatoma cells. Chem. Biol. Interact..

[77] Peterson J.J., Beecher G.R., Bhagwat S.A., Dwyer J.T., Gebhardt S.E., Haytowitz D.B., Holden J.M. (2006). Flavanones in grapefruit, lemons, and limes: A compilation and review of the data from the analytical literature. J. Food Compos. Anal..

[78] Rangel-Huerta O.D., Aguilera C.M., Martin M.V., Soto M.J., Rico M.C., Vallejo F., Tomas-Barberan F., Perez-de-la-Cruz A.J., Gil A., Mesa M.D. (2015). Normal or high polyphenol concentration in orange juice affects antioxidant activity, blood pressure, and body weight in obese or overweight adults. J. Nutr..

[79] Nandakumar N., Balasubramanian M.P. (2012). Hesperidin a citrus bioflavonoid modulates hepatic biotransformation enzymes and enhances intrinsic antioxidants in experimental breast cancer rats challenged with 7, 12-dimethylbenz (a) anthracene. J. Exp. Ther. Oncol..

[80] Mahmoud A.M., Mohammed H.M., Khadrawy S.M., Galaly S.R. (2017). Hesperidin protects against chemically induced hepatocarcinogenesis via modulation of Nrf2/ARE/HO-1, PPARγ and TGF-β1/Smad3 signaling, and amelioration of oxidative stress and inflammation. Chem. Biol. Interact..

[81] Carballo-Villalobos A.I., González-Trujano M.E., Alvarado-Vázquez N., López-Muñoz F.J. (2017). Pro-inflammatory cytokines involvement in the hesperidin antihyperalgesic effects at peripheral and central levels in a neuropathic pain model. Inflammopharmacology.

[82] Akiyama S., Katsumata S., Suzuki K., Ishimi Y., Wu J., Uehara M. (2010). Dietary hesperidin exerts hypoglycemic and hypolipidemic effects in streptozotocin-induced marginal type 1 diabetic rats. J. Clin. Biochem. Nutr..

[83] Mahmoud A.M., Ashour M.B., Abdel-Moneim A., Ahmed O.M. (2012). Hesperidin and naringin attenuate hyperglycemia-mediated oxidative stress and proinflammatory cytokine production in high fat fed/streptozotocin-induced type 2 diabetic rats. J. Diabetes Complicat..

[84] El-Marasy S.A., Abdallah H.M., El-Shenawy S.M., El-Khatib A.S., El-Shabrawy O.A., Kenawy S.A. (2014). Anti-depressant effect of hesperidin in diabetic rats. Can. J. Physiol. Pharmacol..

[85] Visnagri A., Kandhare A.D., Chakravarty S., Ghosh P., Bodhankar S.L. (2014). Hesperidin, a flavanoglycone attenuates experimental diabetic neuropathy via modulation of cellular and biochemical marker to improve nerve functions. Pharm. Biol..

[86] Li W., Kandhare A.D., Mukherjee A.A., Bodhankar S.L. (2018). Hesperidin, a plant flavonoid accelerated the cutaneous wound healing in streptozotocin-induced diabetic rats: Role of TGF-ß/Smads and Ang-1/Tie-2 signaling pathways. EXCLI J..

[87] Dokumacioglu E., Iskender H., Sen T.M., Ince I., Dokumacioglu A., Kanbay Y., Erbas E., Saral S. (2018). The effects of hesperidin and quercetin on serum tumor necrosis factor-alpha and interleukin-6 levels in streptozotocin-induced diabetes model. Pharmacogn. Mag..

[88] Tilg H., Hotamisligil G.S. (2006). Nonalcoholic fatty liver disease: Cytokine-adipokine interplay and regulation of insulin resistance. Gastroenterology.

[89] Mehta S., Farmer J.A. (2007). Obesity and inflammation: A new look at an old problem. Curr. Atheroscler. Rep..

[90] Somerset S.M., Johannot L. (2008). Dietary flavonoid sources in Australian adults. Nutr. Cancer.

[91] Furtado A.F., Nunes M.A., Ribeiro M.H. (2012). Hesperidinase encapsulation towards hesperitin production targeting improved bioavailability. J. Mol. Recognit..

[92] Paramita P., Sethu S.N., Subhapradha N., Ragavan V., Ilangovan R., Balakrishnan A., Srinivasan N., Murugesan R., Moorthi A. (2020). Neuro-protective effects of nano-formulated hesperetin in a traumatic brain injury model of *Danio rerio*. Drug Chem. Toxicol..

[93] Kottaiswamy A., Kizhakeyil A., Padmanaban A., Bushra F., Vijay V.R., Lee P.S., Verma N.K., Kalaiselvan P., Samuel S. (2020). The citrus flavanone hesperetin induces apoptosis in CTCL cells via STAT3/Notch1/NFκB-mediated signaling axis. Anticancer Agents Med. Chem..

[94] Jo S.H., Kim M.E., Cho J.H., Lee Y., Lee J., Park Y.D., Lee J.S. (2019). Hesperetin inhibits neuroinflammation on microglia by suppressing inflammatory cytokines and MAPK pathways. Arch. Pharm. Res..

[95] Kim W.J., Lee S.E., Park Y.G., Jeong S.G., Kim E.Y., Park S.P. (2019). Antioxidant hesperetin improves the quality of porcine oocytes during aging *in vitro*. Mol. Reprod. Dev..

[96] Chen X., Wei W., Li Y., Huang J., Ci X. (2019). Hesperetin relieves cisplatin-induced acute kidney injury by mitigating oxidative stress, inflammation and apoptosis. Chem. Biol. Interact..

[97] Jayaraman R., Subramani S., Sheik Abdullah S.H., Udaiyar M. (2018). Antihyperglycemic effect of hesperetin, a citrus flavonoid, extenuates hyperglycemia and exploring the potential role in antioxidant and antihyperlipidemic in streptozotocin-induced diabetic rats. Biomed. Pharmacother..

[98] Samie A., Sedaghat R., Baluchnejadmojarad T., Roghani M. (2018). Hesperetin, a citrus flavonoid, attenuates testicular damage in diabetic rats via inhibition of oxidative stress, inflammation, and apoptosis. Life Sci..

[99] Fernandes A.A., Novelli E.L., Okoshi K., Okoshi M.P., Di Muzio B.P., Guimarães J.F., Fernandes Junior A. (2010). Influence of rutin treatment on biochemical alterations in experimental diabetes. Biomed. Pharmacother..

[100] Fallahi F., Roghani M., Moghadami S. (2012). Citrus flavonoid naringenin improves aortic reactivity in streptozotocin-diabetic rats. Indian J. Pharmacol..

[101] Hasanein P., Fazeli F. (2014). Role of naringenin in protection against diabetic hyperalgesia and tactile allodynia in male Wistar rats. J. Physiol. Biochem..

[102] Jia S., Hu Y., Zhang W., Zhao X., Chen Y., Sun C., Li X., Chen K. (2015). Hypoglycemic and hypolipidemic effects of neohesperidin derived from *Citrus aurantium* L. in diabetic KK-A(y) mice. Food Funct..

[103] Malakul W., Pengnet S., Kumchoom C., Tunsophon S. (2018). Naringin ameliorates endothelial dysfunction in fructose-fed rats. Exp. Ther. Med..

[104] Alam M.A., Subhan N., Rahman M.M., Uddin S.J., Reza H.M., Sarker S.D. (2014). Effect of citrus flavonoids, naringin and naringenin, on metabolic syndrome and their mechanisms of action. Adv. Nutr..

[105] Chen R., Qi Q.L., Wang M.T., Li Q.Y. (2016). Therapeutic potential of naringin: An overview. Pharm. Biol..

[106] Kandhare A.D., Raygude K.S., Ghosh P., Ghule A.E., Bodhankar S.L. (2012). Neuroprotective effect of naringin by modulation of endogenous biomarkers in streptozotocin induced painful diabetic neuropathy. Fitoterapia.

[107] Ahmad S.F., Attia S.M., Bakheet S.A., Zoheir K.M.A., Ansari M.A., Korashy H.M., Abdel-Hamied H.E., Ashour A.E., Abd-Allah A.R.A. (2015). Naringin attenuates the development of carrageenan-induced acute lung inflammation through inhibition of NF-κb, STAT3 and pro-inflammatory mediators and enhancement of IκBα and anti-inflammatory cytokines. Inflammation.

[108] Jiao H.Y., Su W.W., Li P.B., Liao Y., Zhou Q., Zhu N., He L. (2015). Therapeutic effects of naringin in a guinea pig model of ovalbumin-induced cough-variant asthma. Pulm. Pharmacol. Ther..

[109] Xiong G., Liu S., Gao J., Wang S. (2016). Naringin protects ovalbumin-induced airway inflammation in a mouse model of asthma. Inflammation.

[110] Zhang Y.S., Li Y., Wang Y., Sun S.Y., Jiang T., Li C., Cui S.X., Qu X.J. (2016). Naringin, a natural dietary compound, prevents intestinal tumorigenesis in Apc (Min/+) mouse model. J. Cancer Res. Clin. Oncol..

[111] Kwatra M., Jangra A., Mishra M., Sharma Y., Ahmed S., Ghosh P., Kumar V., Vohora D., Khanam R. (2016). Naringin and sertraline ameliorate doxorubicin-induced behavioral deficits through modulation of serotonin level and mitochondrial complexes protection pathway in rat hippocampus. Neurochem. Res..

[112] Sachdeva A.K., Kuhad A., Chopra K. (2014). Naringin ameliorates memory deficits in experimental paradigm of Alzheimer’s disease by attenuating mitochondrial dysfunction. Pharmacol. Biochem. Behav..

[113] Vij G., Gupta A., Chopra K. (2009). Modulation of antigen-induced chronic fatigue in mouse model of water immersion stress by naringin, a polyphenolic antioxidant. Fundam. Clin. Pharmacol..

[114] Chtourou Y., Aouey B., Kebieche M., Fetoui H. (2015). Protective role of naringin against cisplatin induced oxidative stress, inflammatory response and apoptosis in rat striatum via suppressing ROS-mediated NF-κB and P53 signaling pathways. Chem. Biol. Interact..

[115] Zhang J., Sun C., Yan Y., Chen Q., Luo F., Zhu X., Li X., Chen K. (2012). Purification of naringin and neohesperidin from Huyou (*Citrus* changshanensis) fruit and their effects on glucose consumption in human HepG2 cells. Food Chem..

[116] Gong N., Zhang B., Yang D., Gao Z., Du G., Lu Y. (2015). Development of new reference material neohesperidin for quality control of dietary supplements. J. Sci. Food Agric..

[117] Hwang S.-L., Yen G.-C. (2008). Neuroprotective effects of the citrus flavanones against H2 O2 -induced cytotoxicity in PC12 cells. J. Agric. Food Chem..

[118] Bellocco E., Barreca D., Laganà G., Leuzzi U., Tellone E., Ficarra S., Kotyk A., Galtieri A. (2009). Influence of l-rhamnosyl-d-glucosyl derivatives on properties and biological interaction of flavonoids. Mol. Cell. Biochem..

[119] Zhao T., Hu S., Ma P., Che D., Liu R., Zhang Y., Wang J., Li C., Ding Y., Fu J. (2019). Neohesperidin suppresses IgE-mediated anaphylactic reactions and mast cell activation via Lyn-PLC-Ca(^2+^) pathway. Phytother. Res..

[120] Guo J., Fang Y., Jiang F., Li L., Zhou H., Xu X., Ning W. (2019). Neohesperidin inhibits TGF-β1/Smad3 signaling and alleviates bleomycin-induced pulmonary fibrosis in mice. Eur. J. Pharmacol..

[121] Tan Z., Cheng J., Liu Q., Zhou L., Kenny J., Wang T., Lin X., Yuan J., Quinn J.M.W., Tickner J. (2017). Neohesperidin suppresses osteoclast differentiation, bone resorption and ovariectomised-induced osteoporosis in mice. Mol. Cell. Endocrinol..

[122] Jiang C.H., Sun T.L., Xiang D.X., Wei S.S., Li W.Q. (2018). Anticancer activity and mechanism of xanthohumol: A prenylated flavonoid from hops (*Humulus lupulus* L.). Front. Pharmacol..

[123] Li F., Yao Y., Huang H., Hao H., Ying M. (2018). Xanthohumol attenuates cisplatin-induced nephrotoxicity through inhibiting NF-κB and activating Nrf2 signaling pathways. Int. Immunopharmacol..

[124] Wang S., Yang C., Tu H., Zhou J., Liu X., Cheng Y., Luo J., Deng X., Zhang H., Xu J. (2017). Characterization and metabolic diversity of flavonoids in *Citrus* species. Sci. Rep..

[125] Sharma A., Sharma P., Singh Tuli H., Sharma A.K. (2018). Phytochemical and Pharmacological Properties of Flavonols. eLS.

[126] Gautam R., Singh M., Gautam S., Rawat J.K., Saraf S.A., Kaithwas G. (2016). Rutin attenuates intestinal toxicity induced by Methotrexate linked with anti-oxidative and anti-inflammatory effects. BMC Complement. Altern. Med..

[127] Klimczak I., Małecka M., Szlachta M., Gliszczyńska-Świgło A. (2007). Effect of storage on the content of polyphenols, vitamin C and the antioxidant activity of orange juices. J. Food Compos. Anal..

[128] Stuetz W., Prapamontol T., Hongsibsong S., Biesalski H. (2010). Polymethoxylated flavones, flavanone glycosides, carotenoids, and antioxidants in different cultivation types of tangerines (*Citrus reticulata* Blanco cv. Sainampueng) from Northern Thailand. J. Agric. Food Chem..

[129] Ramful D., Bahorun T., Bourdon E., Tarnus E., Aruoma O.I. (2010). Bioactive phenolics and antioxidant propensity of flavedo extracts of Mauritian citrus fruits: Potential prophylactic ingredients for functional foods application. Toxicology.

[130] Gattuso G., Barreca D., Gargiulli C., Leuzzi U., Caristi C. (2007). Flavonoid composition of citrus juices. Molecules.

